# Supplementary material for: Asymmetric Ru-In atomic pairs promote highly active and stable acetylene hydrochlorination
Source: Nat Commun. 2024 Jul 17;15:6035. doi: 10.1038/s41467-024-50221-3 (PMC11254904; doi:10.1038/s41467-024-50221-3)
Supplement: Supplementary file 1 — Supplementary Information [file 41467_2024_50221_MOESM1_ESM.pdf]

Supplementary Information

Asymmetric Ru-In Atomic Pairs Promote Highly Active and Stable Acetylene Hydrochlorination

Yurui Fan[1,#], Haomiao Xu[1,#,*], Guanqun Gao[1], Mingming Wang[1], Wenjun Huang[1], Lei Ma[1], Yancai Yao[1,*], Zan Qu[1,*], Pengfei Xie[2,*], Bin Dai[3], and Naiqiang Yan[1,4]

[1]School of Environmental Science and Engineering, Shanghai Jiao Tong University, Shanghai 200240, China

[2]College of Chemical and Biological Engineering, Zhejiang University, Hangzhou 310058, China

[3]State Key Laboratory Incubation Base for Green Processing of Chemical Engineering, School of Chemistry and Chemical Engineering, Shihezi University, Shihezi 832003, China

[4]Shanghai Institute of Pollution Control and Ecological Security, Shanghai 200092, China

*Corresponding authors: Dr. Haomiao Xu, Dr. Yancai Yao, Dr. Zan Qu, and Dr. Pengfei Xie

E-mail: *xuhaomiao@sjtu.edu.cn*, *yyancai@sjtu.edu.cn*, *quzan@sjtu.edu.cn*, and *pfxie@zju.edu.cn*

[#]Y. R. Fan and H. M. Xu contributed equally to this work.

Experimental Section

1 Chemicals

Aniline (C_6H_7N), Ruthenium(III) acetylacetonate ($Ru(acac)_3$), indium nitrate hydrate ($In(NO_3)_3 \cdot 5H_2O$), 1,4-dicarboxybenzene (H_2BDC) ($C_8H_6O_4$), N,N-Dimethylformamide (DMF), methanol (CH_3OH), ethanol (CH_3CH_2OH), acetone (C_3H_6O), ammonium persulphate ($(NH_4)_2S_2O_8$), NaOH, H_2SO_4, and HCl were provided by Sinopharm Chemical Reagent Co., Ltd. (Shanghai, China). Commercial activated carbon (CAC) was purchased from Meryer Chemical Technology Co., Ltd. (Shanghai, China). The HCl gas (> 99 %) and C_2H_2 gas (> 99 %) were obtained from Liquefaction Air Gas Co., Ltd. (Shanghai, China). All the reagents, without special instruction, were employed without further purification. All solutions were prepared with deionized (DI) water (>18.2 MΩ).

2 Catalyst characterization

Powder X-ray diffraction (XRD) patterns were acquired using a powder X-ray diffractometer (PANalytical, Netherlands) with Cu-Kα radiation (λ = 1.54060 Å) over the 2θ range from 10 to 80° and at a scan rate of 2°/min.

Laser Raman spectra were obtained on a LabRAM HR800 Evolutions (Horiba, Japan) spectrometer in the range of 400–3000 cm^{-1} by employing an excitation wavelength of 532 nm line with Ar ion laser.

Fourier transform infrared (FTIR) spectroscopy was recorded using a Nicolet 6700 FT-IR spectrometer, which the spectra were recorded at every certain time by accumulating 64 scans with a resolution of 4 cm^{-1}.

The chemical composition and relative contents (the elemental concentrations were quantified based on the measured photoelectron peak areas) of the catalyst samples were acquired by X-ray photoelectron spectroscopy (XPS) equipped with Al-Kα radiation (Thermo Fischer, ESCALAB 250Xi), in which the all data were calibrated with C 1s binding energy at 284.8 eV.

Inductively coupled plasma-optical emission spectrometry (ICP-OES) was carried out on an Agient ICP-OES 5110 instrument. The sample (10 mg) was added into the digestion tank containing 7 mL mixed acid solution (V(HCl): V(HNO_3) = 2 : 1) and 0.75 mL HF solution, followed by digestion treatment at 500 °C for several hours until the solid material had fully dissolved.

The catalyst morphology was characterized by transmission electron microscopy (TEM), and the corresponding energy-dispersive X-ray spectroscopy (EDS) images were also obtained. The high-resolution transmission electron microscopy (HR-TEM) and aberration-corrected high-angle annular dark-field-scanning scanning transmission electron microscopy (AC-HAADF-STEM) were performed on a double-corrected microscope JEM-ARM200F (GrandARM, JEOL).

N_2 adsorption-desorption isotherms (Barrett-Joyner-Halenda (BJH) method) and Brunaur-Emmett-Teller (BET) surfaces areas were acquired with a surface area analyzer (Quantachrome NOVA 2200E).

Thermogravimetry (TG) and analysis was carried out from 50 to 800 °C by using a Mettler Toledo in an air atmosphere with a heating rate of 10 °C/min.

Temperature-programmed desorption (TPD) experiments were measured from 30 to 600 °C with 10 °C/min heating rate by a Quantachrome Instruments (AMI-90) at He atmosphere.

H_2-TPR experiment was performed on an automatic adsorption instrument (AutoChem II 2920), in which the catalysts were firstly degassed at 300 °C for 3 h under Ar atmosphere, and then reduced at 10% H_2/Ar mixing atmosphere from 30 to 500 °C.

Soxhlet extraction was used to confirm the recyclability and reusability of catalyst. Briefly, the fresh catalyst was extracted in acetone solution at 120 °C for 48 h, where this process should always maintain the cooling water refluxing to prevent the escape of acetone. Then, approximately 0.5 mL of Ru extraction solution was quantified, and the rest part was reloaded onto the fresh supports using the same procedures in Figure 1a.

X-ray absorption near edge structure (XANES) and extended X-ray absorption fine structure (EXAFS) measurements of Ru and In were collected at the beamline BL14W1 of the Shanghai Synchrotron Radiation Facility (SSRF, Shanghai) in a fluorescence mode at room temperature. Data reduction, data analysis, and EXAFS fitting were performed and analyzed with the Athena and Artemis programs of the Demeter data analysis packages that utilizes the FEFF6 program to fit the EXAFS data. The energy calibration of the sample was conducted through a standard Ru foil and In foil, which as a reference was simultaneously measured. A linear function was subtracted from the pre-edge region, then the edge jump was normalized using Athena software. The $\chi(k)$ data were isolated by subtracting a smooth, third-order polynomial approximating the absorption background of an isolated atom. The k^3-weighted $\chi(k)$ data were Fourier transformed after applying Hanning window function ($\Delta k = 1.0$). For EXAFS modeling, The global amplitude EXAFS (CN, R, σ^2 and ΔE_0) were obtained by nonlinear fitting, with least-squares refinement, of the EXAFS equation to the Fourier-transformed data in R-space, using Artemis software, EXAFS of the Ru foil and In foil are fitted and the obtained amplitude reduction factor S_0^2 value (0.863 and 0.788) was set in the EXAFS analysis to determine the coordination numbers (*CNs*) in the Ru/In-N scattering path in sample.

In-situ FT-IR spectra of C_2H_2 adsorption was conducted on a Nicolet 6700 FT-IR spectrometer using a liquid N_2-cooled mercury cadmium telluride (MCT-A) detector. The samples (~5 mg) were gently pressed onto KBr pellet and placed into a cell. Then, the samples were pretreated in flowing N_2 (50 mL/min) at 300 °C for 1 h and then cooled to 25 °C, during which the background spectra were recorded. Then the feed gas were switched to C_2H_2 gas and kept at 25 °C for 0.5 h, and spectra were recorded at every certain time. Spectra were recorded at every certain time by accumulating 64 scans with a resolution of 4 cm^{-1}. The intensity of adsorbed species was evaluated in Kubelka-Munk Units (KMU), derived from the reflectance (R_v) through the equation of $KMU\nu = (1-R_v)^2/2R_v$ that was proved to be proportional to the adsorbate concentration over a wide range of experimental conditions.

In-situ FT-IR spectra of NH_3 adsorption was recorded on a Nicolet 6700 spectrometer in the range of 600~2000 cm^{-1} with a resolution of 4 cm^{-1} and 64 scans. Briefly, 30 mg of catalyst powder was put in an in situ IR cell equipped with the KBr window. The catalyst was pre-treated with N_2 at 300 °C for 1 h. Then the cell was cooled down to 100 °C with N_2 flow (100 mL/min). The catalyst itself was used as the background of the spectra. Following this, 5% NH_3/N_2 (50 mL/min) gas was introduced into the cell and adsorbed for 45 min at 100 °C, where it was observed that the bands associated with NH_3 adsorption on catalysts no longer changed. The system was flushed for 30 min in N_2 with 100 mL/min to remove any gas-phase NH_3, and then the spectra were preserved.

3 Simulation details

All the spin-polarized density functional theory calculations were conducted by using the Vienna ab initio simulation package (VASP). The D3 correction approach (DFT-D3) was employed in order to reflect the impact of van der Waals (vdW) interactions. The exchange-functional is treated using the generalized gradient approximation (GGA) of Perdew-Burke-Ernzerhof (PBE) functional. The projector-augmented wave (PAW) approach was used to signify core-valence interactions. Electron smearing was employed through the Gaussian smearing method with a smearing width of 0.05 eV. The energy cutoff for the plane wave basis expansion was set to 500 eV. Optimized structures were obtained by minimizing the forces on each ion using the conjugate gradient algorithm until they are below 0.02 eV/Å. The self-consistent calculations apply a convergence energy threshold of 10^{-5} eV. The Monkhorst-Pack K-mesh for the Brillouin-zone integration with K-point separation of 0.04 $Å^{-1}$ were applied for the calculation.

The reaction energy (ΔE) and activation (E_a) energy were calculated by the Born-Oppenheimer energy difference between IS, TS, and FS:

$$\Delta E = E_{FS} - E_{IS} \quad (1)$$

$$E_a = E_{TS} - E_{IS} \quad (2)$$

where E_{IS}, E_{TS}, and E_{FS} are the energies of IS, TS, and FS, respectively, and the equation

for calculating E_{ads} is given, as follows:

$$E_{ads} = E_{total} - E_{substrate} - E_{adsorbate} \quad (3)$$

where E_{total}, $E_{substrate}$, and $E_{adsorbate}$ are the total energies of the substrate and adsorbate, the energy of the substrate, and that of the adsorbate, respectively.

Charge density difference was obtained by the following equation:

$$\Delta\rho = \rho_{AB} - \rho_{A} - \rho_{B} \quad (4)$$

where $\Delta\rho$ represents the charge of the total system, and ρA and ρB represent the charge of segment A and segment B, respectively.

The free energy of adsorbates was calculated from the harmonic vibrational energies hvi using VASPKIT (v.1.2.0) according to the following formulas:

$$F = U - T \times S \quad (5)$$

$$U = E_{DFT} + E_{ZPE} + U(T) \quad (6)$$

$$S = \sum_i \left\{ \frac{\frac{hv_i}{kT}}{e^{\frac{hvi}{kT}} - 1} - \ln\left[1 - e^{\frac{hvi}{kT}}\right] \right\} \quad (7)$$

$$U(T) = \sum_i \frac{\frac{hv_i}{k}}{e^{\frac{hvi}{k}} - 1} \quad (8)$$

where F, U, T, S, E_{DFT}, E_{ZPE}, $U(T)$ and v_i represent free energy, internal energy, temperature, entropy, DFT energy, zero point energy, correction of heat capacity and vibrational frequencies, respectively. The h is Planck constant and k is Boltzmann constant.

Supplementary Text 1

Internal Diffusion: Weisz-Prater Criterion

The absence of internal mass transfer limitations was evaluated using the Weisz-Prater criterion , where if C_{WP} is lower than 1, the internal mass transfer effects can be neglected:

$$C_{wp} = \frac{-r_{A(obs)}\rho R^2}{D_e C_{AS}} \tag{9}$$

where $-r_{A(obs)}$ = Observed reaction rate (1.98×10^{-4} kmol/s/kg$_{cat.}$)

ρ = Solid catalyst density (750 kg/m^3)

R = Particle radius (6.25×10^{-5} m)

C_{AS} = Concentration of reaction gas at the surface of the catalyst. (1.4×10^{-2} kmol/m^3)

D_e = Effective gas-phase diffusity = $\frac{D_{AB}\varepsilon_p\sigma_c}{\tau}$ (10)

D_{AB} = Gas-phase diffusivity. D_{AB} for a mixture was calculated according to Perry's Chemical Engineer's Handbook to be 4.13×10^{-5} m^2/s

ε_p = Pellet porosity = 0.5

σ_c = Constriction factor = 0.5

τ = Tortuosity = 3

$D_e = 0.34\times10^{-5}$ m^2/s

Thus, the C_{wp} value was calculated to be 0.012 (< 1), indicating this system does not suffer from internal mass transfer limitations.

Supplementary Text 2

External Diffusion: Mears Criterion

The absence of external mass transfer limitations can be evaluated using the Mears criterion:

$$\frac{-r_{A(obs)}\rho_b R n}{k_c C_{Ab}} \tag{11}$$

where $-r_{A(obs)}$ = observed reaction rate. (1.98×10^{-4} kmol/s/kg_{cat})

ρ_b = Bulk density of the catalyst bed. (396 kg/m^3)

R = Particle radius. (6.25×10^{-5} m)

n = Reaction order If n = 2

k_c = Mass transfer coefficient. (0.14 m/s)

C_{Ab} = Concentration of reaction gas at the surface of the catalyst. (1.4×10^{-2} kmol/m^3)

Thus, the Mears Criterion was calculated to be 0.005 (< 0.15), which demonstrated that this system does not suffer from external mass transfer limitations.

Supplementary Table 1 The fresh and used Ru/In loading, as well as the loss amount of Ru/In of Ru-N-In/NC, RuN_4/NC, and InN_4/NC catalysts, determined by ICP-OES.

Catalyst	Ru loading (wt.%)	ΔRu (wt.%)	In loading (wt.%)	ΔIn (wt.%)
Ru-N-In/NC-0 h	0.978	/	0.187	/
Ru-N-In/NC-100 h	0.972	0.006	0.186	0.001
Ru-N-In/NC-200 h	0.822	0.150	0.183	0.003
Ru-N-In/NC-300 h	0.820	0.002	0.182	0.001
Ru-N-In/NC-400 h	0.819	0.001	0.181	0.001
Ru-N-In/NC-500 h	0.819	0	0.180	0.001
Ru-N-In/NC-600 h	0.818	0.001	0.180	0
RuN_4/NC-0 h	0.984	/	/	/
RuN_4/NC-100 h	0.942	0.042	/	/
InN_4/NC-0 h	/	/	0.988	/
InN_4/NC-100 h	/	/	0.983	0.005

ΔRu and ΔIn represent the loss amount of Ru or In every 100 hours.

Supplementary Table 2 Pore structure parameters of the as-prepared Ru-based catalysts.

Catalyst	BET surface area (m^2/g)	Total pore volume (cm^3/g)	Average pore diameter (nm)
NC600	802	0.37	2.68
NC700	930	0.40	2.52
NC800	1020	0.29	2.30
NC900	910	0.39	2.71
NC1000	753	0.34	3.39
RuN_4/NC800	678	0.35	3.69
InN_4/NC800	634	0.37	3.19
Ru-N-In/NC800	690	0.43	2.72

Supplementary Table 3 EXAFS fitting parameters at the Ru and In *K*-edge for various samples.

Samples	Coordination	CN^a	R(Å)^b	σ^2(Å^2)^c	ΔE_0(eV)^d	R factor
Ru foil	Ru-Ru	12*	2.672±0.002	0.0040±0.0005	-3.2±0.8	0.0038
**Ru**-N-In/NC	Ru-N	3.5±0.3	1.989±0.012	0.0101±0.0012	-1.5±2.0	0.0043
**Ru**N_4/NC	Ru-N	3.9±0.3	2.042±0.004	0.0034±0.0011	-2.0±1.2	0.0062
In foil	In-In	12*	3.249±0.021	0.0241±0.0023	1.8±0.5	0.0074
Ru-N-**In**/NC	In-N	4.1±0.3	2.134±0.005	0.0112±0.0016	2.4±1.5	0.0102

$^a CN$, coordination number; $^b R$, the distance to the neighboring atom; $^c \sigma^2$, the mean square relative displacement (MSRD); $^d \Delta E_0$, inner potential correction; R factor indicates the goodness of the fit. S_0^2 was fixed to 0.863 and 0.788, according to the experimental EXAFS fit of Ru foil and In foil by fixing CN as the known crystallographic value. * This value was fixed during EXAFS fitting, based on the known structure of Ru and In. Fitting range: $3.0 \le k$ (/Å) ≤ 13.5 and $1.0 \le R$ (Å) ≤ 3.0 (Ru foil); $3.0 \le k$ (/Å) ≤ 10.5 and $1.0 \le R$ (Å) ≤ 2.5 (**Ru**-N-In/NC); $3.0 \le k$ (/Å) ≤ 12.5 and $1.0 \le R$ (Å) ≤ 2.5 (**Ru**N_4/NC); $3.0 \le k$ (/Å) ≤ 11.0 and $2.0 \le R$ (Å) ≤ 4.0 (In foil); $3.0 \le k$ (/Å) ≤ 10.5 and $1.0 \le R$ (Å) ≤ 2.5 (Ru-N-**In**/NC). A reasonable range of EXAFS fitting parameters: $0.700 < S_0^2 < 1.000$; $CN > 0$; $\sigma^2 > 0$ Å^2; $|\Delta E_0| < 10$ eV; R factor < 0.02.

Revised Supplementary Table 4 EXAFS fitting parameters at the Ru and In *K*-edge for Ru-N-In/NC.

Sample	Coordination	*CN*	*R*(Å)	σ^2(Å^2)	ΔE_0(eV)	*R* factor
Ru-N-In/NC	Ru-N	3.4±0.4	2.008±0.011	0.0043±0.0019	0.2±1.6	0.0014
	Ru-N-Ru/In	1.7±0.5	3.610±0.017	0.0102±0.0049	-8.5±0.5	
	In-N	4.1±0.7	2.130±0.006	0.0056±0.0016	0.9±0.3	0.0013
	In-N-In/Ru	1.8±0.6	3.608±0.028	0.0132±0.0059	-9.6±2.1	

Note that *CN*: coordination numbers; R: bond distance; σ^2: Debye-Waller factors; ΔE_0: the inner potential correction; *R* factor: goodness of fit. A reasonable range of EXAFS fitting parameters: $0.700 < S_0^2 < 1.000$; $CN > 0$; $\sigma^2 > 0$ Å^2; $|\Delta E_0| < 10$ eV; *R* factor < 0.02. Fitting range: $3.0 \leq k$ (/Å) ≤ 13.5 and $1.0 \leq R$ (Å) ≤ 3.0 (Ru foil); $3.0 \leq k$ (/Å) ≤ 10.5 and $1.0 \leq R$ (Å) ≤ 4.0 (**Ru**-N-In/NC); $3.0 \leq k$ (/Å) ≤ 11.0 and $2.0 \leq R$ (Å) ≤ 4.0 (In foil); $3.0 \leq k$ (/Å) ≤ 10.5 and $1.0 \leq R$ (Å) ≤ 4.0 (Ru-N-**In**/NC).

Supplementary Table 5 Catalytic performances of the Ru-based catalysts in acetylene hydrochlorination recently reported in literature.

No.	Catalysts	Reaction conditions			C_2H_2 conversion	TOS (h)	Deactivation rate (%/h)
		T/°C	*GHSV* $(C_2H_2)/h^{-1}$	V(HCl)/ $V(C_2H_2)$			
1	Ru@TPPB/AC[1]	180	180	1.15	99.7%	48	0.032
2	Ru-L_8/AC[2]	180	180	1.15	99.0%	24	0.025
3	Ru-L_1/AC[2]	180	180	1.15	99.0%	24	0.057
4	Ru-O/AC-O[3]	180	180	1.15	99.0%	24	0.333
5	Ru[BMIM]BF_4/AC[4]	180	180	1.15	98.9%	24	0.029
6	Φ-P-Ru/AC-HNO_3[5]	180	180	1.15	97.2%	48	0.212
7	TPAP-Ru/AC-HCl[6]	180	180	1.15	95.0%	48	0.125
8	(Ru/AC)[7]	180	180	1.15	95.0%	48	0.135
9	(Ru/AC)-N_2[7]	180	180	1.15	91.1%	24	0.271
10	$(NH_4)_2RuCl_6$/AC[8]	180	180	1.15	90.5%	48	0.067
11	CuRu/MWCNTs[9]	180	180	1.15	51.6%	65	0.077
12	$CuCl_2$ V-N SAC[10]	180	180	1.15	/	60	0.082
13	RuCu(0)/AC[11]	180	180	1.15	81.4%	48	0.131
14	RuCu(I)/AC[11]	180	180	1.15	94.0%	48	0.018

15	RuCu(II)/AC[11]	180	180	1.15	96.9%	48	0.015
16	Ru-NC[12]	180	180	1.15	85.18%	50	0.343
17	Ru-NC@CAU[12]	180	180	1.15	92.45%	80	0.121
18	Ru-NC@MIL[12]	180	180	1.15	99.03%	150	0.009
19	IPr-Ru/AC[13]	180	180	1.15	99.0%	50	0.029
20	Ru/AC@PEI[14]	180	180	1.15	85.0%	100	0.180
21	1Ru-5IL_5/AC[15]	180	180	1.15	99.0%	200	0.010
22	Ru_5Cl_7/AC[16]	180	180	1.15	96.1%	48	0.322
23	Ru-N-OMC[17]	180	180	1.15	91.8%	30	0.217
24	Ru/AC[18]	180	180	1.15	72.5%	100	0.198
25	Ru/NC-APS[19]	170	90	1.15	96.9%	600	0.02
26	RuN_4/NC[This work]	180	180	1.15	76.35%	120	0.398
27	Ru-N-In/NC[This work]	180	180	1.15	99.58%	600	0.001

Supplementary Table 6 Desorption peak area of the catalysts determined by TPD.

Catalyst	Desorption area of C_2H_2	Desorption area of HCl
InN_4/NC	6653.7	2638.7
RuN_4/NC	2329.7	5474.3
Ru-N-In/NC	5605.5	4467.3

Supplementary Table 7 EXAFS fitting parameters of the Ru *K*-edge for various post-hydrochlorination samples.

Samples	Coordination	CN^a	$R(\text{Å})^b$	$\sigma^2(\text{Å}^2)^c$	$\Delta E_0(\text{eV})^d$	*R* factor
Ru-N-In/NC-3 h	Ru-N	3.2±0.2	1.966±0.003	0.0049±0.0012	5.2±1.3	0.0038
	Ru-Cl	0.9±0.3	2.391±0.014	0.0090±0.0017	3.3±2.1	
RuN_4/NC-1 h	Ru-N	3.1±0.3	2.012±0.008	0.0035±0.0009	1.5±2.3	0.0095
	Ru-Cl	1.3±0.2	2.364±0.021	0.0025±0.0021	3.2±1.6	
RuN_4/NC-3 h	Ru-N	3.2±0.4	2.029±0.005	0.0091±0.0015	4.6±2.8	0.0089
	Ru-Cl	2.6±0.5	2.378±0.011	0.0043±0.0022	1.2±1.6	

$^a CN$, coordination number;

$^b R$, the distance to the neighboring atom;

$^c \sigma^2$, the mean square relative displacement (MSRD);

$^d \Delta E_0$, inner potential correction;

R factor indicates the goodness of the fit.

A reasonable range of EXAFS fitting parameters: $0.700 < S_0^2 < 1.000$; $CN > 0$; $\sigma^2 > 0$ Å^2; $|\Delta E_0| < 10$ eV; R factor < 0.02.

Supplementary Table 8 Fitting parameters derived from Cl 2*p* XPS spectra of the fresh and used Ru/NC and Ru-N-In/NC catalysts.

Catalysts	C-Cl bond		Ru-Cl bond	
	Position (eV)	Area (%)	Position (eV)	Area (%)
RuN_4/NC fresh	200.4	42.73	198.8	57.27
RuN_4/NC-1h	200.4	56.67	198.8	43.33
RuN_4/NC-2h	200.4	59.82	198.8	40.18
RuN_4/NC-3h	200.3	62.35	198.7	37.65
Ru-N-In/NC fresh	200.0	40.92	198.7	59.08
Ru-N-In/NC-1h	200.2	56.01	198.8	43.99
Ru-N-In/NC-2h	200.2	55.79	198.7	44.21
Ru-N-In/NC-3h	200.3	55.10	198.8	44.90

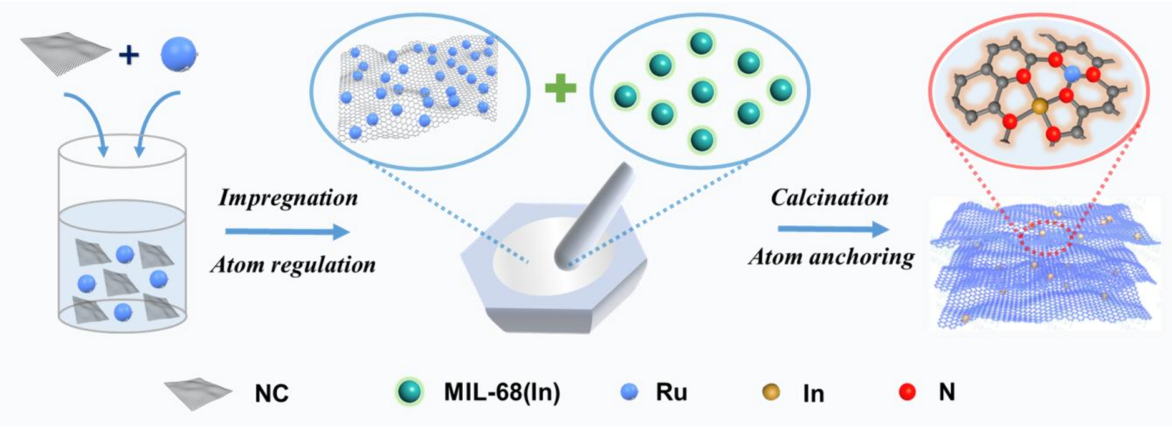

Supplementary Figure 1. Illustration of the synthetic process of the Ru-N-In/NC sample.

Briefly, nitrogenous carbon (NC) powder (the pyrolysis product of polyaniline) and ruthenium acetylacetonate ($Ru(acac)_3$) are dispersed in deionized water to form uniform mixtures (denoted as Ru-NC). Then, this mixture was mixed with MIL-68(In) through homogeneous grinding to obtain catalyst precursors before calcination treatment under the argon atmosphere.

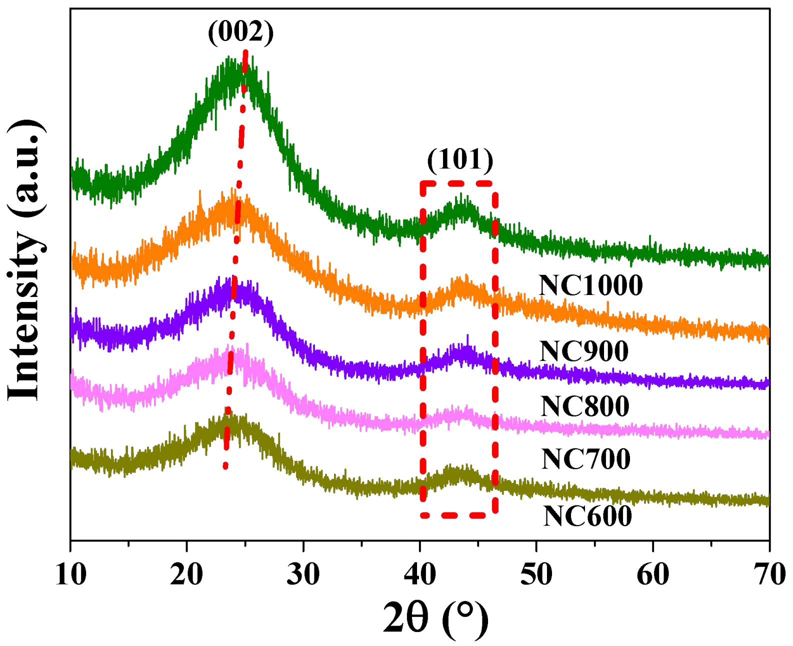

Supplementary Figure 2. XRD patterns of the NC(T_C) samples with different carbonization temperature ranging from 600 to 1000°C.

Supplementary Figure 2 shows the X-ray diffraction (XRD) patterns of the NC(T_C) (T_C = 600, 700, 800, 900, and 1000 °C) samples. Only two broad peaks were found at ~25.8° and ~43.3°, which can be assigned to (002) and (101) planes of graphitic carbon, respectively. No other peaks of the metal oxides or metallic phases were detected for the NC(T_C) samples. Notably, as the increasing of carbonization temperature, the (002) peaks presented a slight shift (from 23.6° to 25.8°), and the intensities of (101) peaks were gradually strengthened, suggesting the graphitization degree of NC(T_C) increased at higher temperatures.

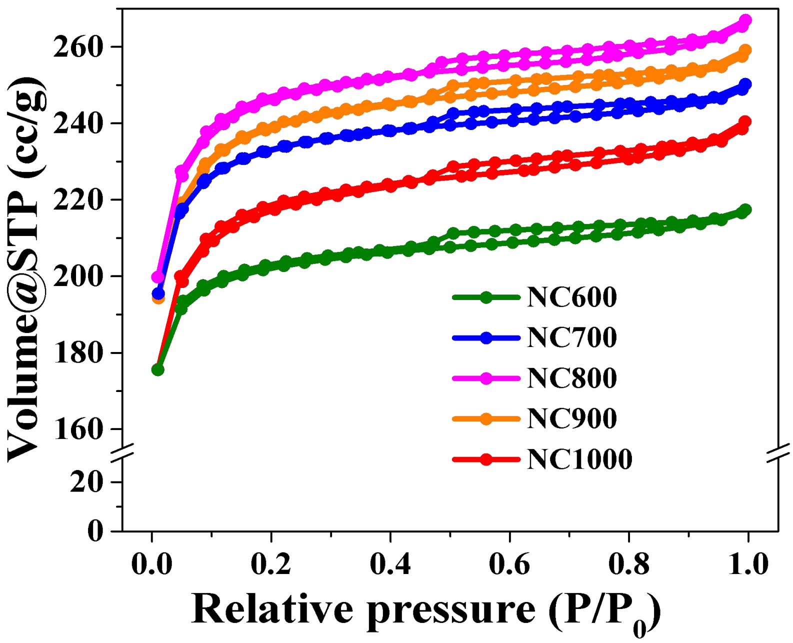

Supplementary Figure 3. N_2 adsorption and desorption curves of the NC samples with different carbonization temperature ranging from 600 to 1000°C.

N_2 adsorption-desorption analysis was applied to investigate the pore characteristics of the NC(T_C) samples, in which the adsorption isotherm curves of all the samples presented Type I/H4 model. The obtained pore structure parameters of each sample are summarized in **Supplementary Table 2**. The pore size distribution analysis confirmed that the average pore diameter of the NC(T_C = 600, 700, 800, and 900) samples actually dominated from 2.2997 to 2.7056 nm, whereas the relatively high total pore volume is helpful to expose the active site and promote the mass transfer of gas molecules. On the contrary, the pore diameter of NC1000 (~3.3931 nm) was bigger than those of other samples with mesoporous structures, which the reason can be attributed to the collapse of microporous structures under the force of excessive high temperature. The BET results show a trend of increasing first and then decreasing with rising of combustion temperature, of which the NC800 catalyst reached the largest specific surface area of 1020.31 m^2/g.

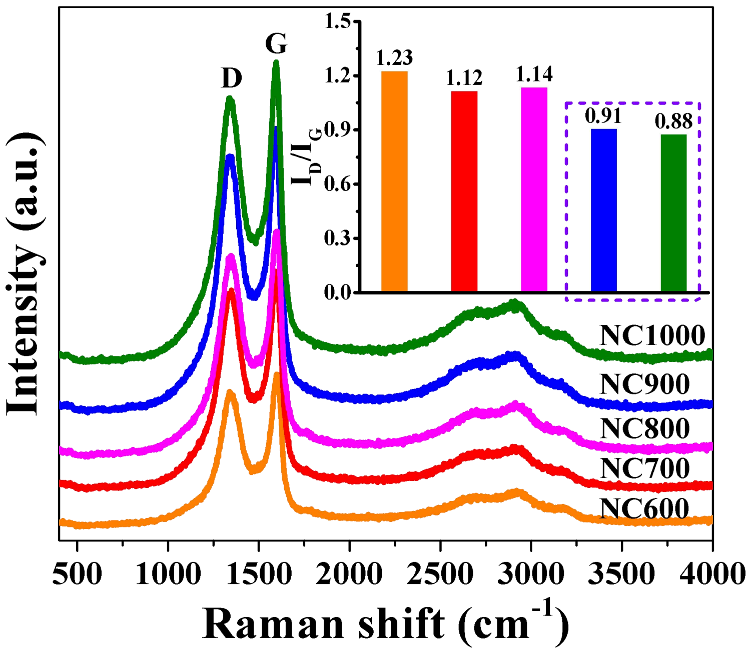

Supplementary Figure 4. Raman spectra of the NC(T_C) samples with different carbonization temperature ranging from 600 to 1000°C.

Raman spectroscopy can provide the thickness and defect information of carbon materials, so as to obtain basic judgments on the characteristics of carbon structure. The D band and G band were separately located at ~1342.8 and 1589.2 cm^{-1}, corresponding to the amorphous structure and graphitized structure of the carbon material surface . The intensity of the G band became significantly strong once the carbonization temperature was higher than 900 °C due to the formation of graphite crystals. The gradually clear peaks at 2684.8 cm^{-1} of NC900 and NC1000 also suggest that monolayer graphene is more likely to appear in carbon with higher graphitization degree. The defects in the carbon matrix could be estimated by I_D/I_G values, which is also used to reflect the graphitic degree. As presented in the inserted figure in **Supplementary Figure 4**, the I_D/I_G values of NC900 (0.91) and NC1000 (0.88) were below 1.0, whereas the rest samples were above 1.1, indicating that more defects were generated before the graphite phase was formed.

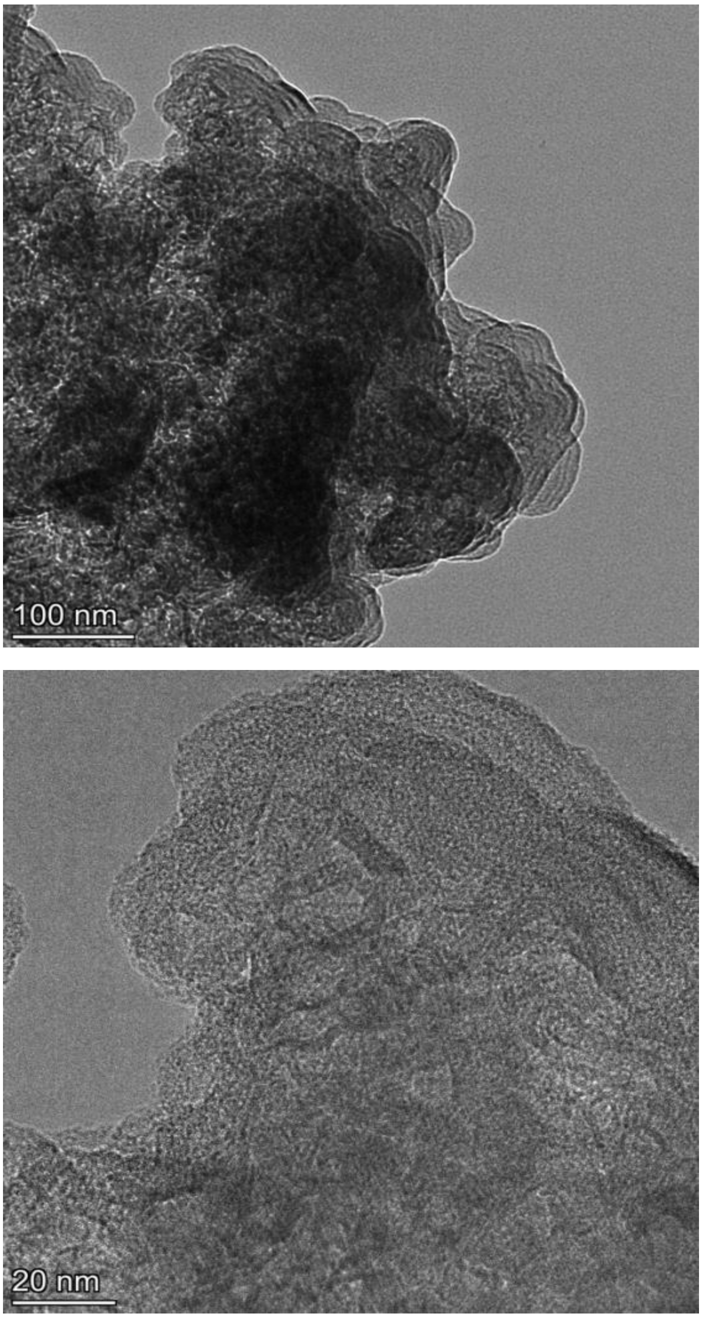

Supplementary Figure 5. TEM images of the NC supports.

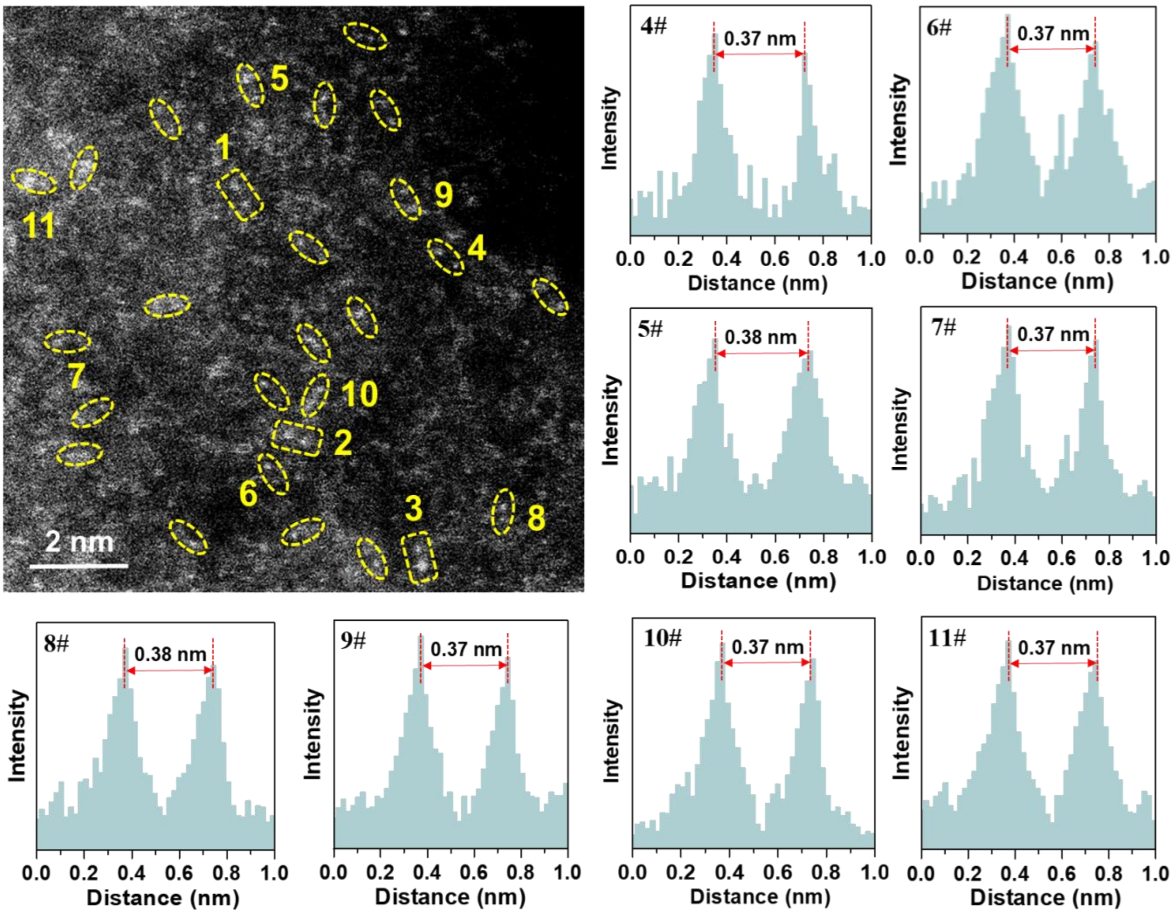

Supplementary Figure 6. AC-HAADF-STEM image of the Ru-N-In/NC sample, and the distance (nm) of isolated Ru and In dual single-atom sites from 4# to 11#.

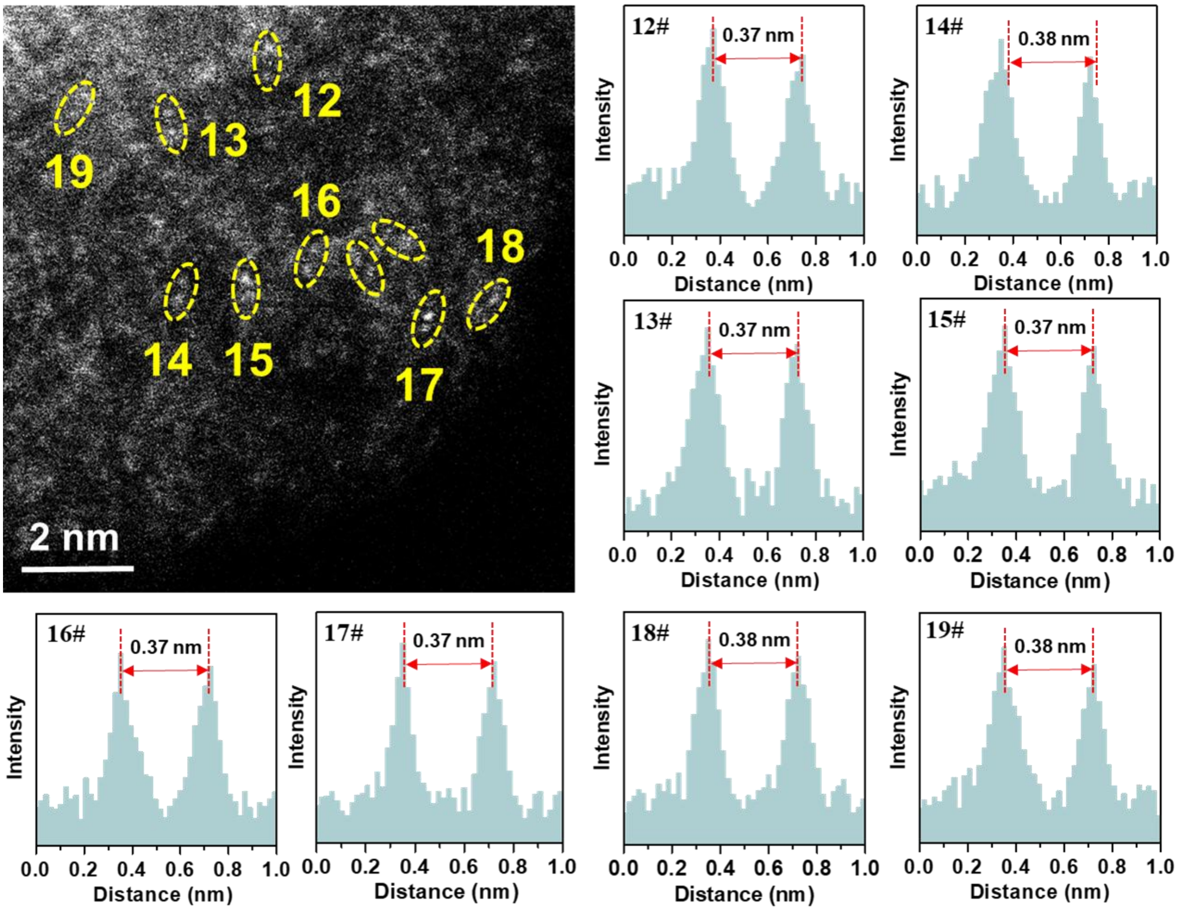

Supplementary Figure 7. AC-HAADF-STEM image of the Ru-N-In/NC sample, and the distance (nm) of isolated Ru and In dual single-atom sites from 12# to 19#.

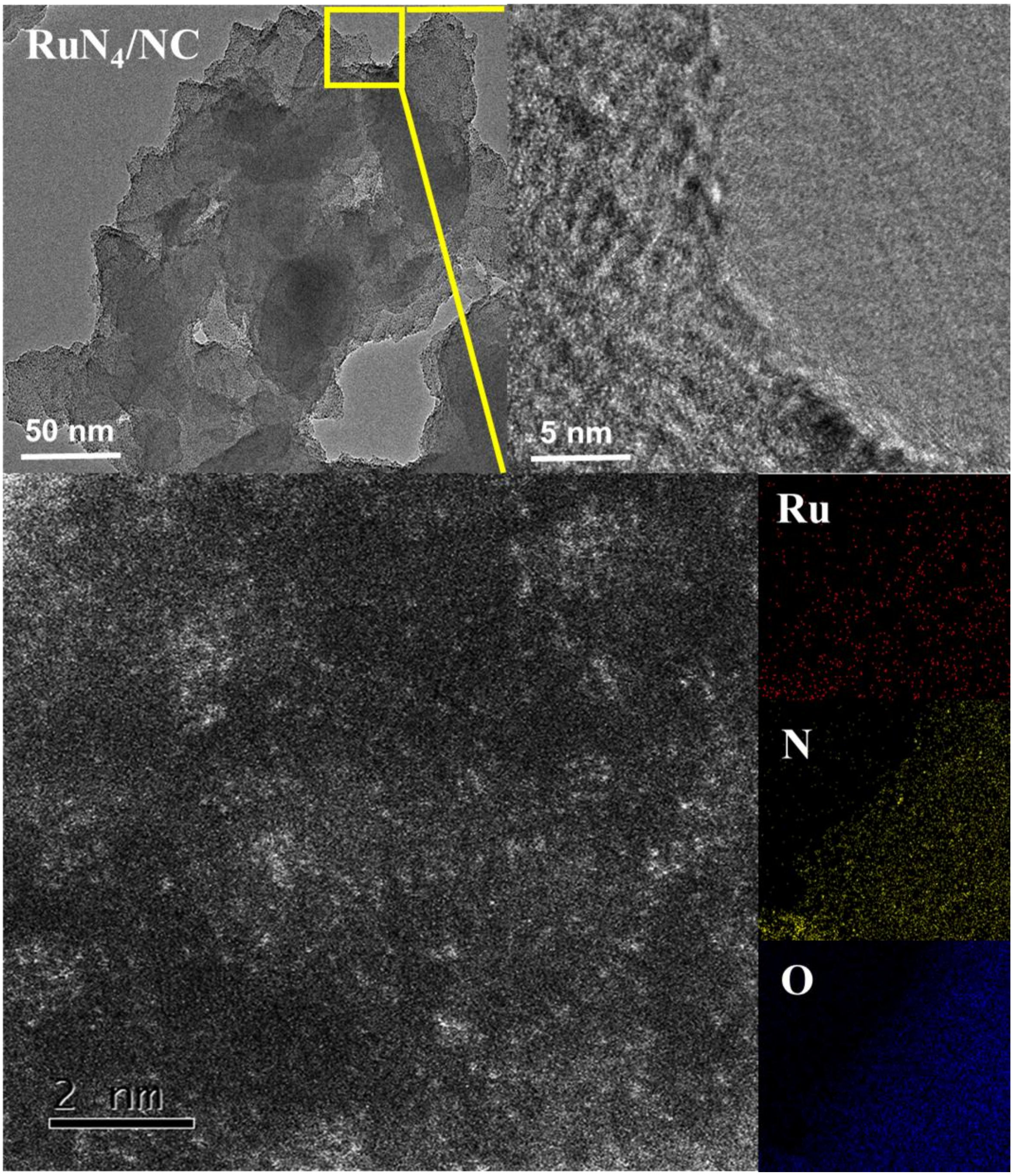

Supplementary Figure 8. TEM, AC-HAADF-STEM, and EDS images for RuN_4/NC.

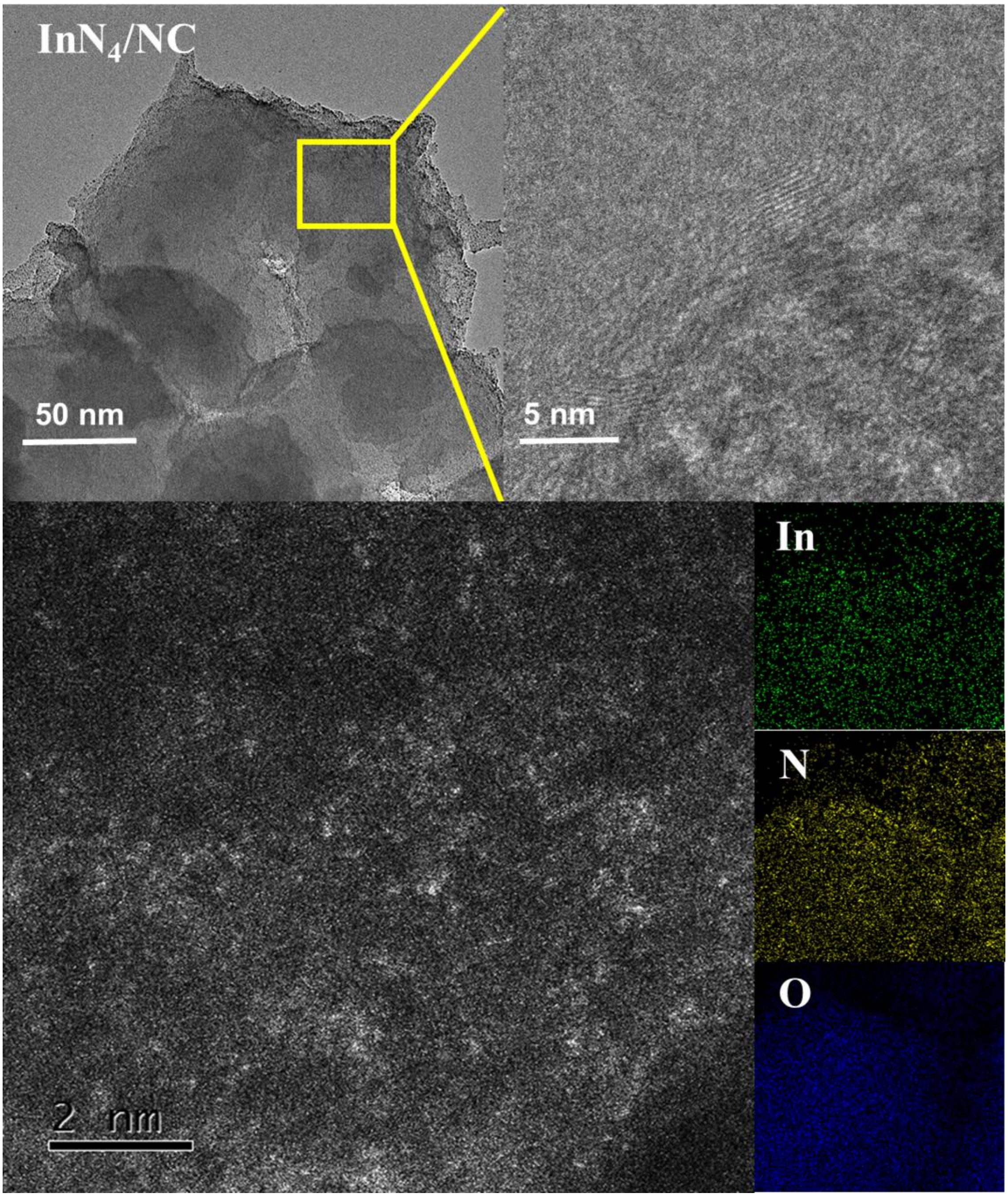

Supplementary Figure 9. TEM, AC-HAADF-STEM, and EDS images for InN_4/NC.

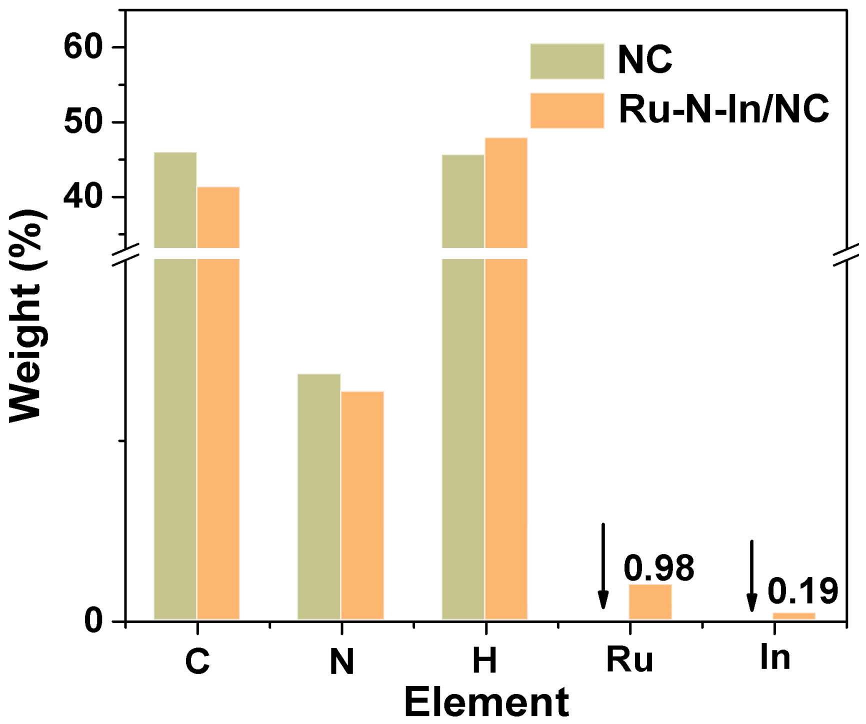

Supplementary Figure 10. Element contents in Ru-N-In/NC and NC obtained by elemental analysis.

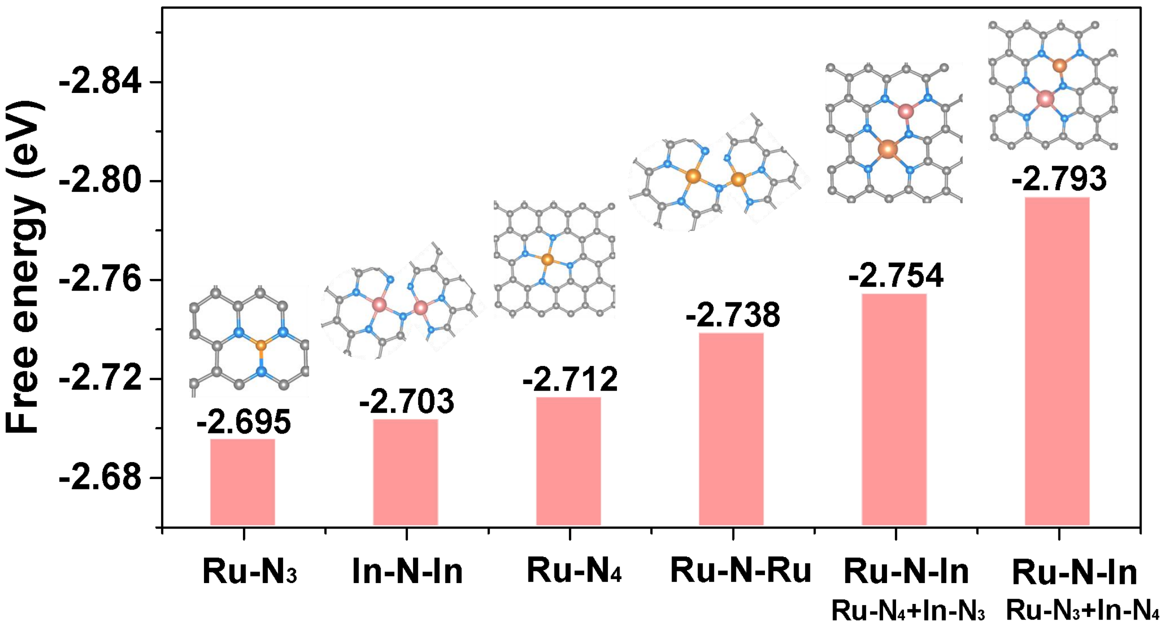

Supplementary Figure 11. Structure of various Ru-based models and their free energies calculated by DFT. Orange, pink, blue, and gray spheres represent Ru, In, N, and C atoms, respectively.

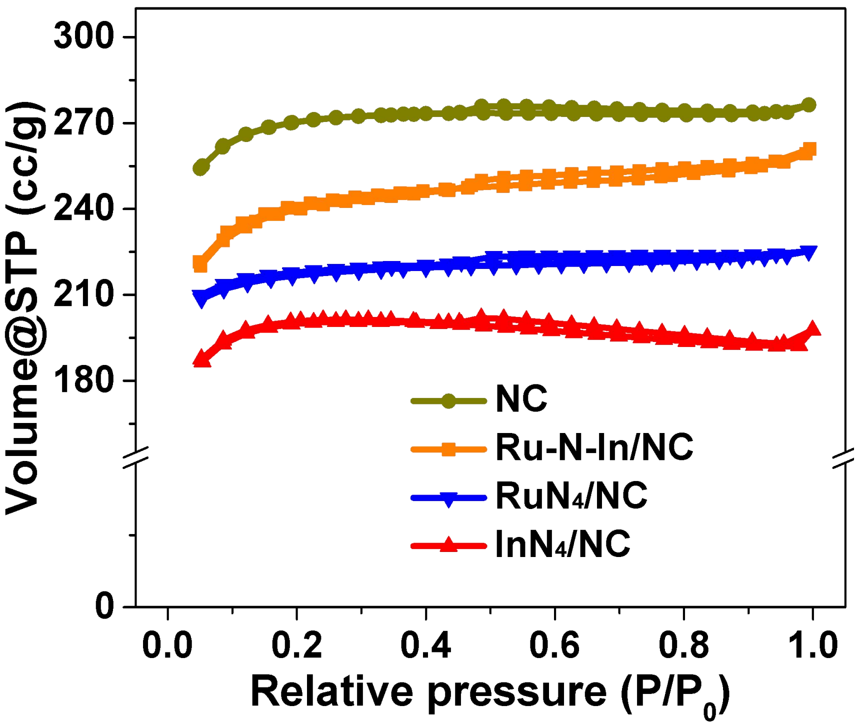

Supplementary Figure 12. N_2 adsorption-desorption curves of Ru-N-In/NC, RuN_4/NC, InN_4/NC, and NC.

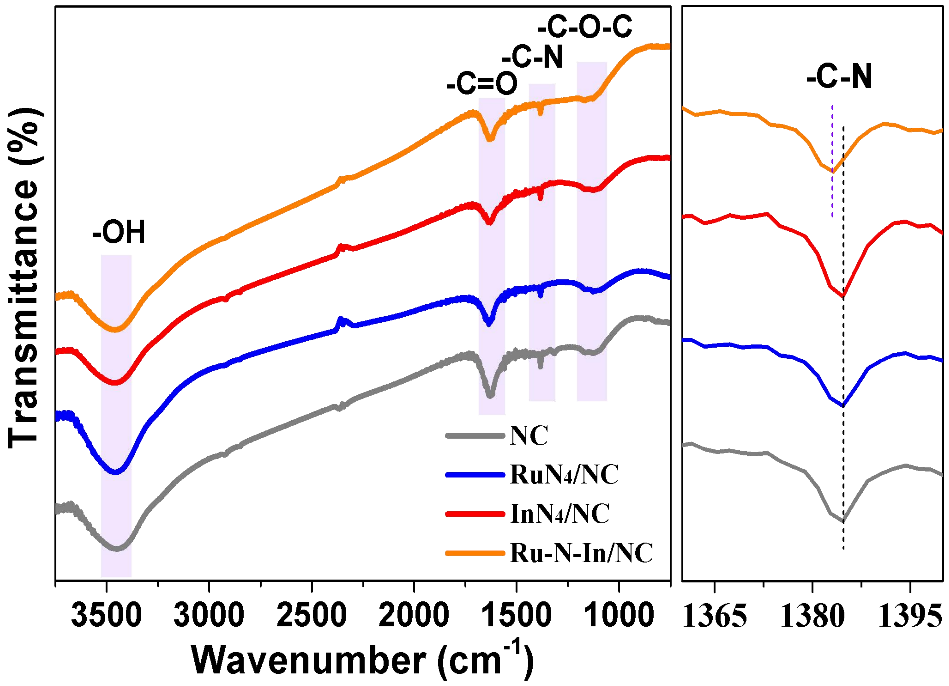

Supplementary Figure 13. FT-IR spectra of Ru-N-In/NC, RuN_4/NC, InN_4/NC, and NC.

All metal/NC samples show the characteristic stretching vibrations similar to that of NC. The broad peak at ~3658 to 3454 cm^{-1} originated from the stretching vibration and deformation vibration of −OH. The bands that appear at 1461 and 1385 cm^{-1} correspond to the sp^2 C=N and sp^3 C–N mode, respectively. Moreover, the band located at around 1684–1622 cm^{-1} can be assigned to the –C=O bonds. Note that the peak of C-N stretching in Ru-N-In/NC was observed a slight blue-shift compared to the RuN_4/NC, InN_4/NC, and NC samples. This indicates that the C-N coordination is affected by Ru-In configuration anchored on NC.

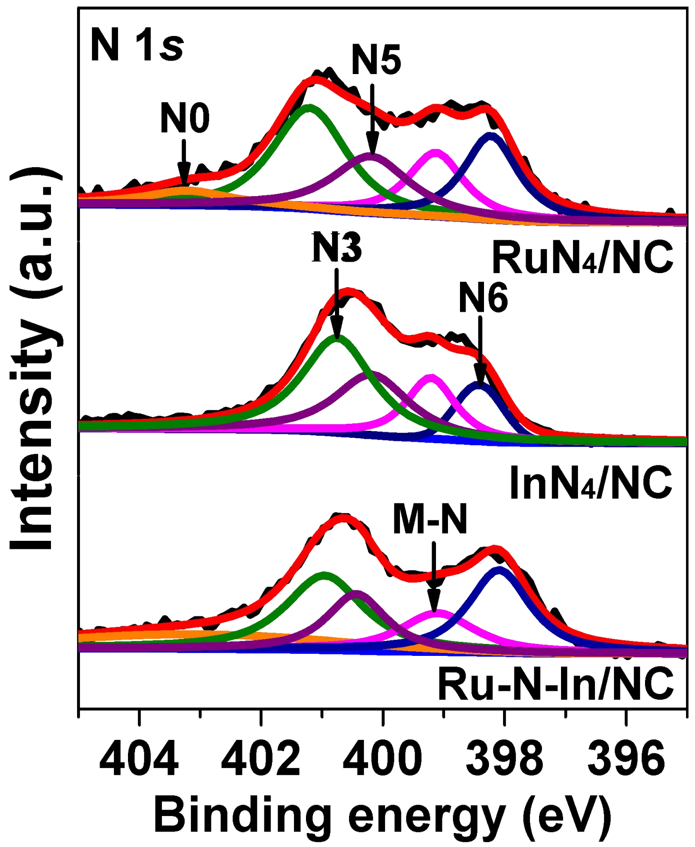

Supplementary Figure 14. N 1s XPS spectra of Ru-N-In/NC, RuN_4/NC, and InN_4/NC. Five distinct N-sites were identified by N 1s XPS, including pyridinic (N6, B.E. = 398 ± 0.2 eV), pyrrolic (N5, B.E. 400.4 ± 0.2 eV), graphitic (N3, B.E. = 401.1 ± 0.2 eV), oxidized nitrogen (N0, B.E. = 403.1 ± 0.2 eV), and M-N (M = Ru or In) species (B.E. = 399.1 ± 0.2 eV). Notably, for Ru-N-In/NC, all the peaks are shifted to the lower the binding energy.

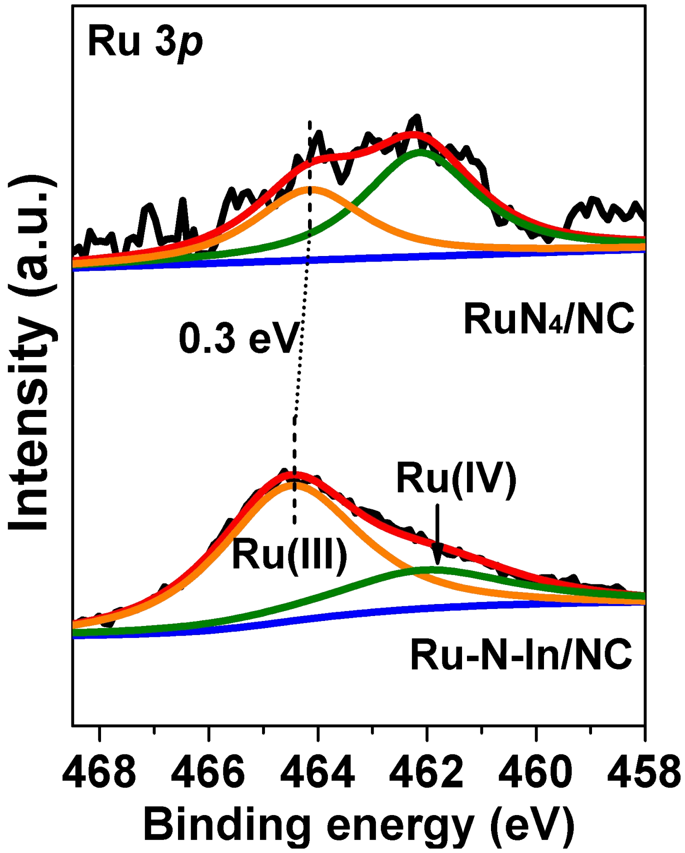

Supplementary Figure 15. Ru 3*p* XPS spectra of Ru-N-In/NC and RuN_4/NC. The introduction of In can stabilize Ru on NC matrix and guarantee the high valence Ru species.

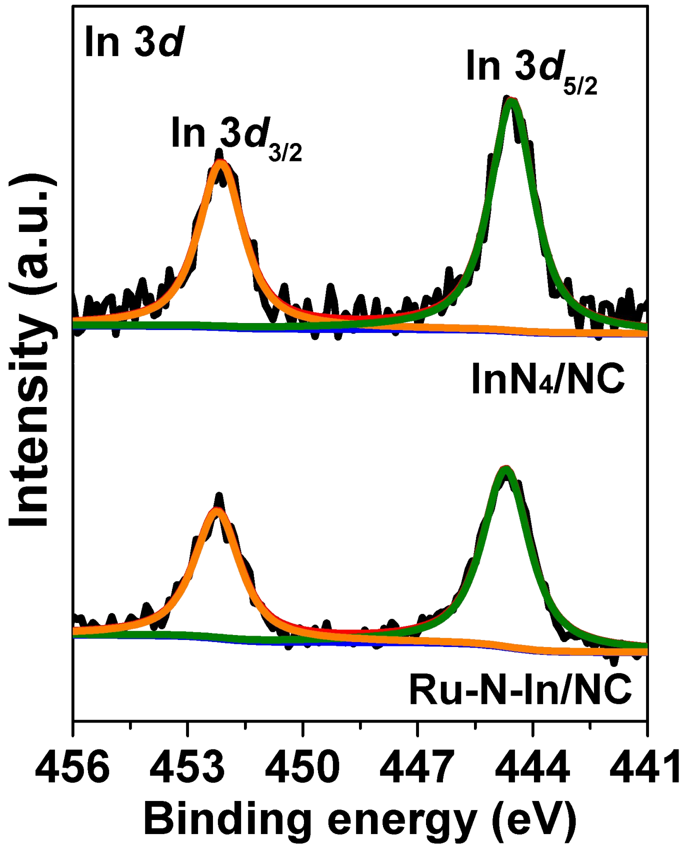

Supplementary Figure 16. In 3*d* XPS spectra of Ru-N-In/NC and InN_4/NC. The binding energy of In 3$d_{3/2}$ and In 3$d_{5/2}$ peaks of InN_4/NC and Ru-N-In/NC are higher than those of the standard In_2O_3 peaks, revealing that the valence state of In is higher than +3.

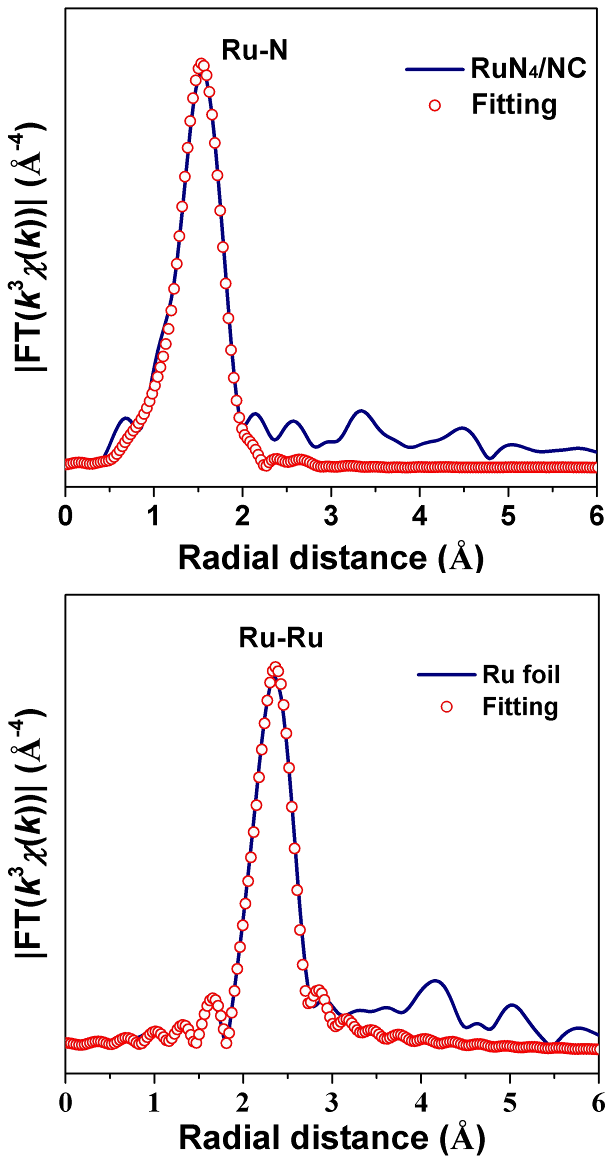

Supplementary Figure 17. EXAFS fitting curves for RuN_4/NC and Ru foil.

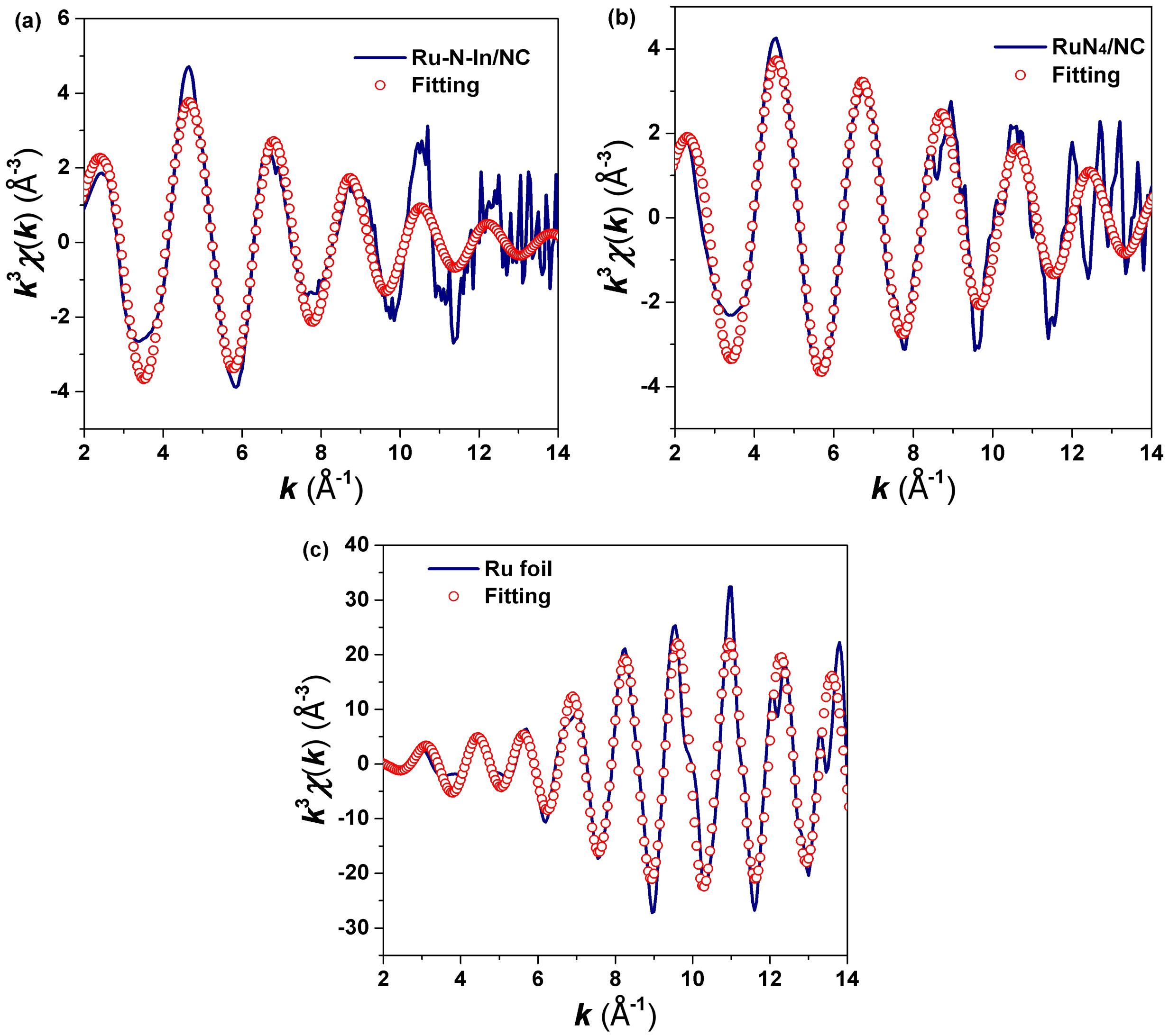

Supplementary Figure 18. The *R* space fitting curves at Ru K-edge of (a) Ru-N-In/NC, (b) RuN_4/NC, and (c) Ru foil.

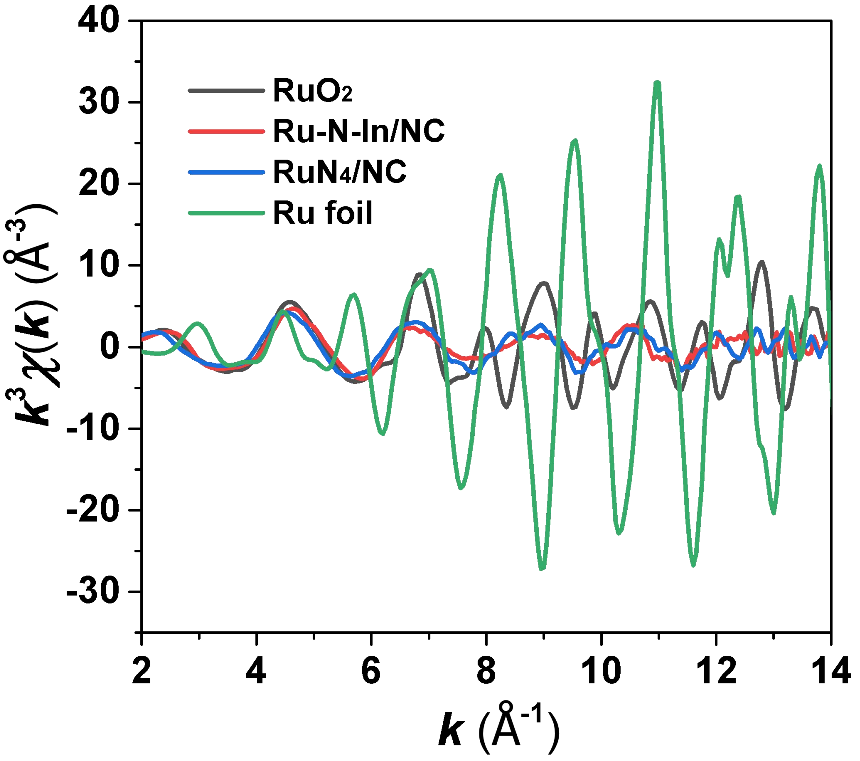

Supplementary Figure 19. EXAFS oscillations at Ru K-edge of Ru-N-In/NC, RuN_4/NC, RuO_2, and Ru foil.

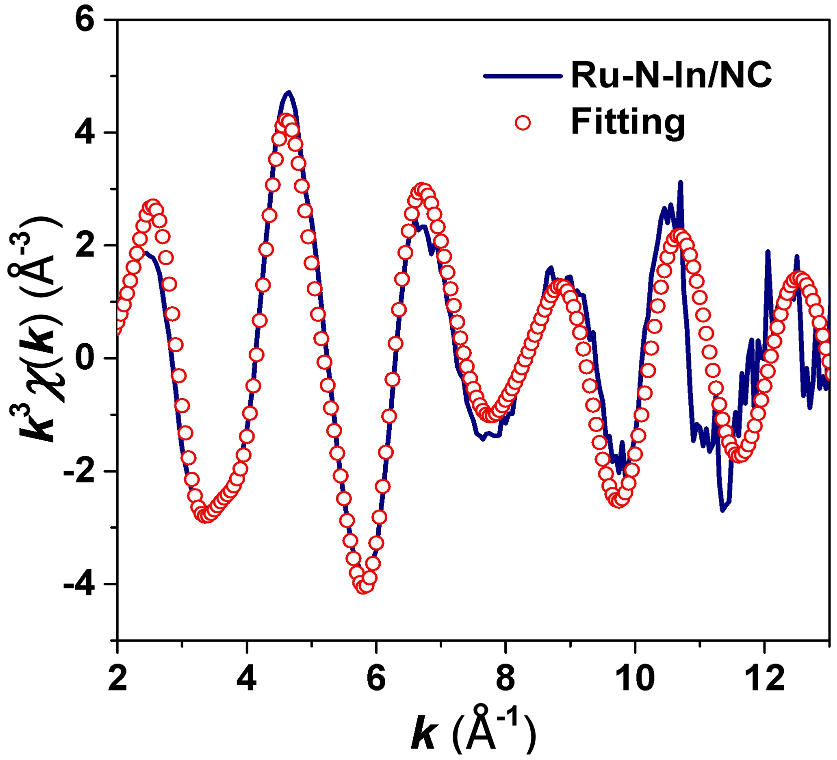

Supplementary Figure 20. The R space fitting curve of Ru in Ru-N-Ru/In.

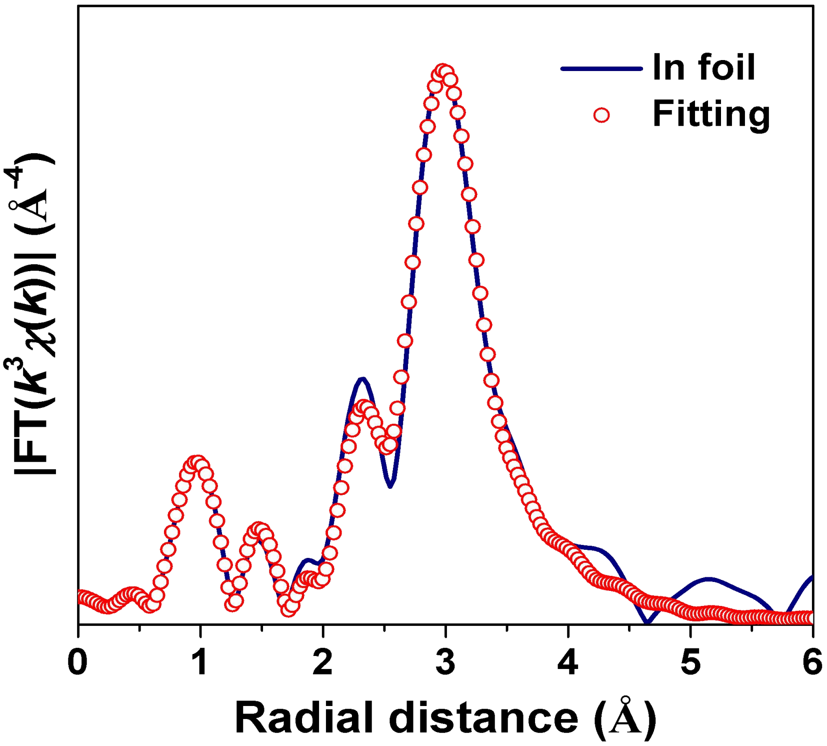

Supplementary Figure 21. EXAFS fitting curves for In foil.

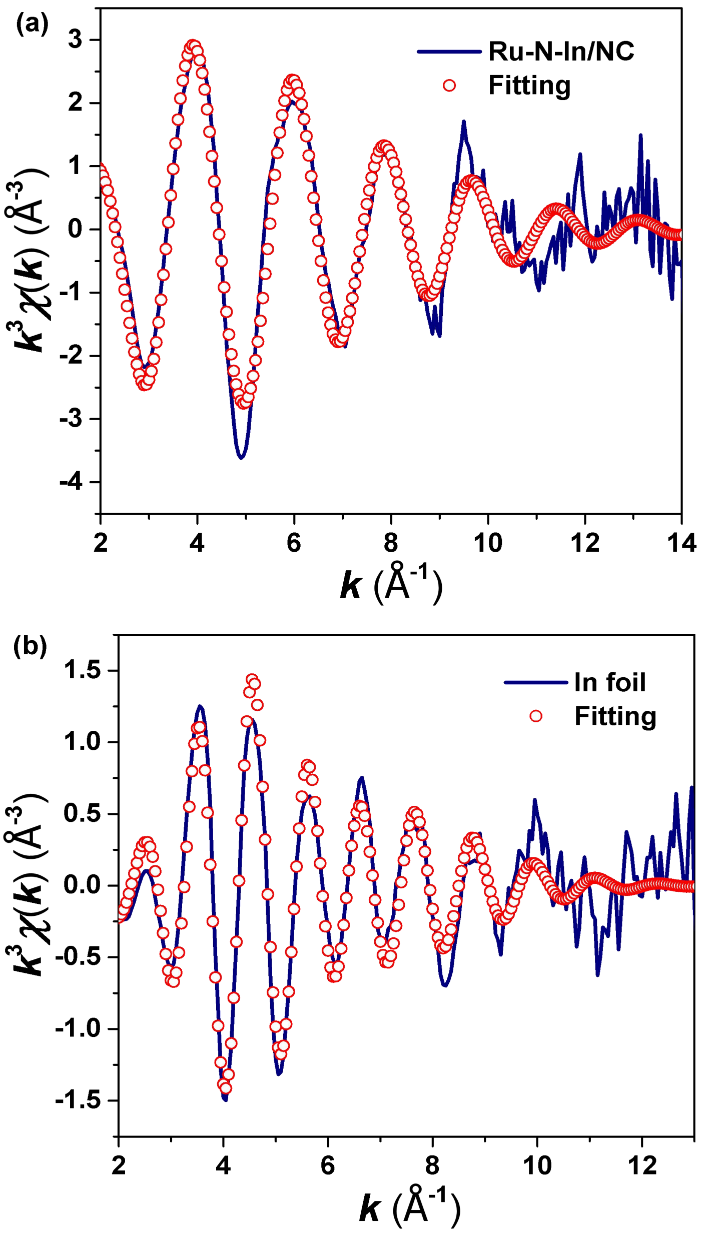

Supplementary Figure S22. The *R* space fitting curves at In K-edge of (a) Ru-N-In/NC, (b) In foil.

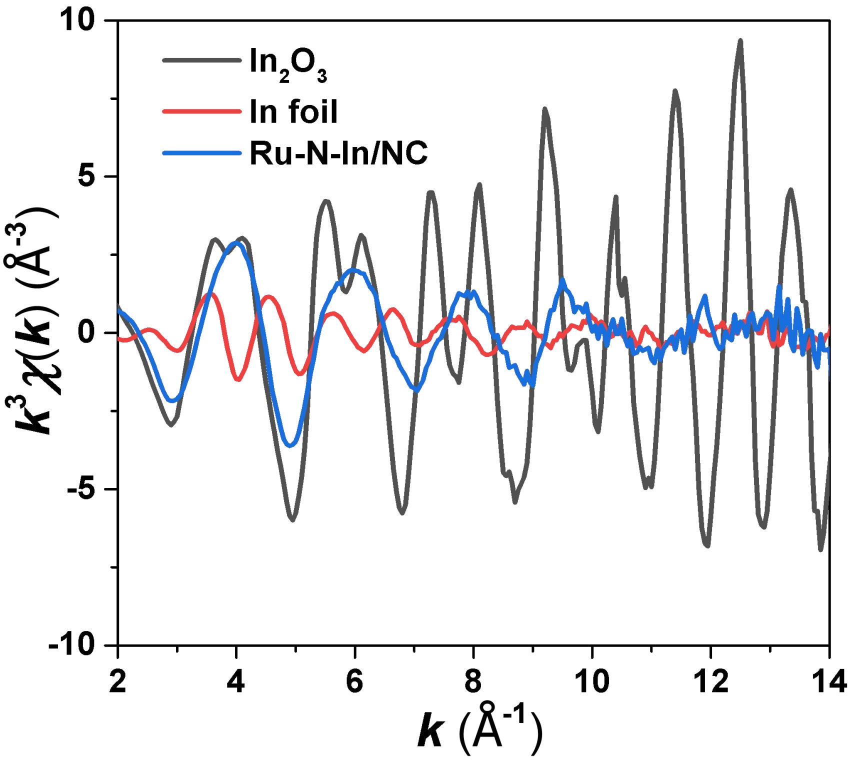

Supplementary Figure 23. EXAFS oscillations at In K-edge of Ru-N-In/NC, In_2O_3, and In foil.

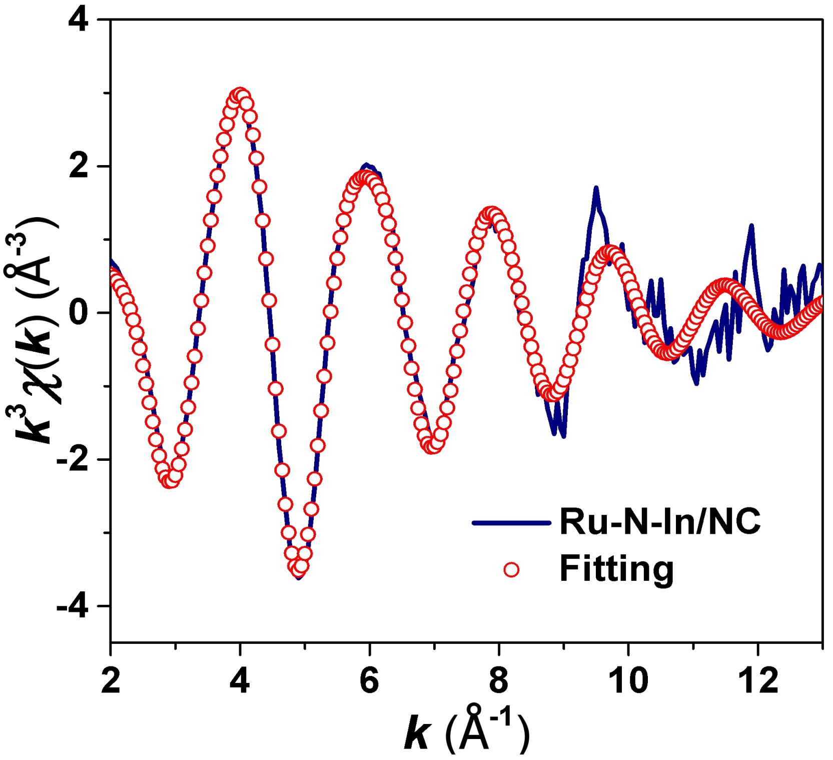

Supplementary Figure 24. The R space fitting curve of In in Ru-N-Ru/In.

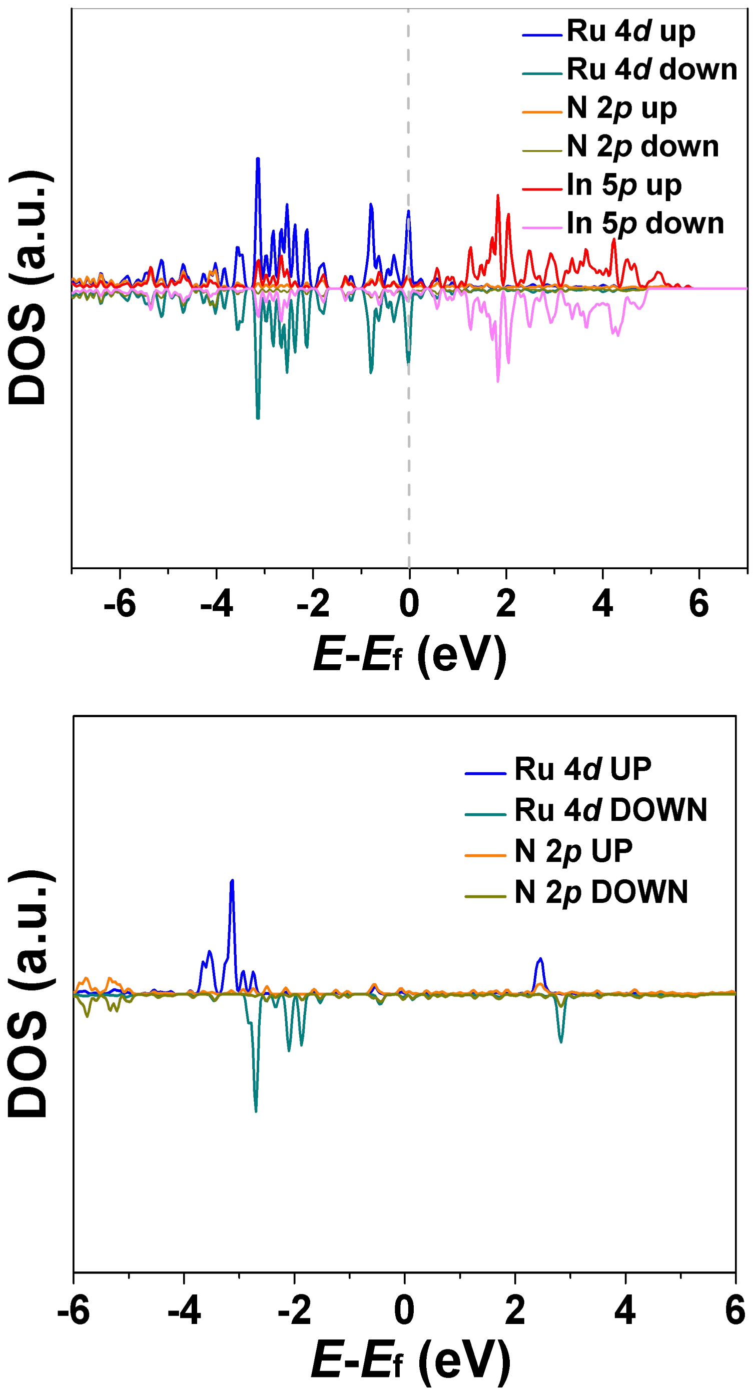

Supplementary Figure 25. Partial density of states (PDOS) of the Ru-N-In/NC and RuN_4/NC configurations.

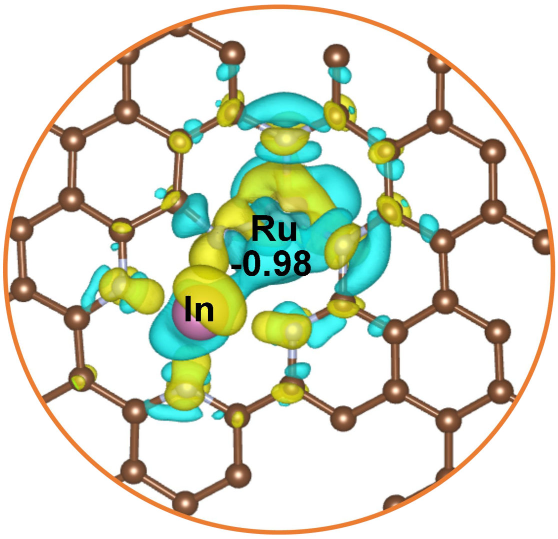

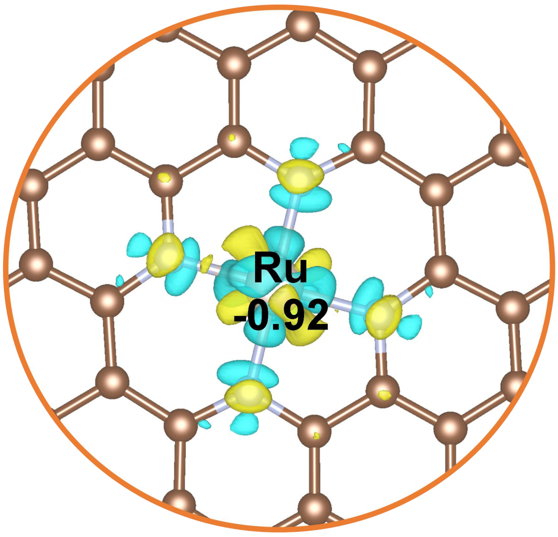

Supplementary Figure 26. Charge-density difference plots of the Ru-N-In/NC and RuN_4/NC configurations (isosurface level is 0.1 e/Å). Note that the yellow and cyan isosurfaces represent charge accumulation and charge depletion, respectively. Orange, pink, blue, and gray spheres represent Ru, In, N, and C atoms, respectively.

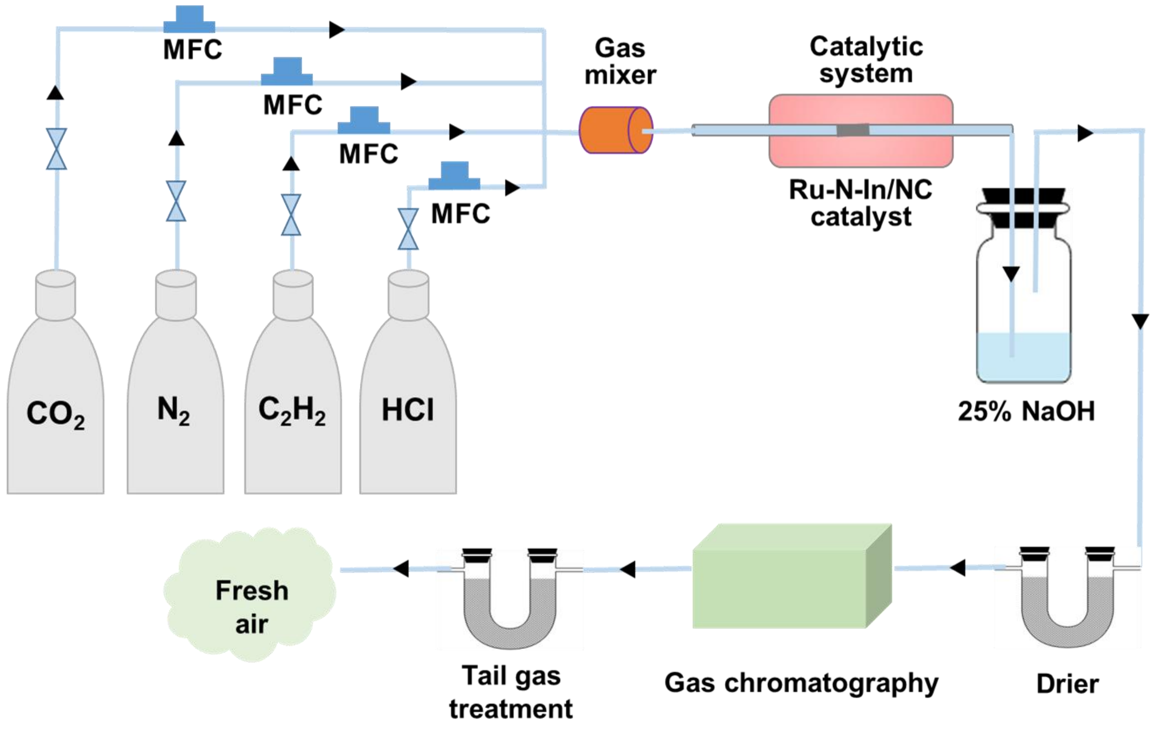

Supplementary Figure 27. Schematic of experimental apparatus for acetylene hydrochlorination.

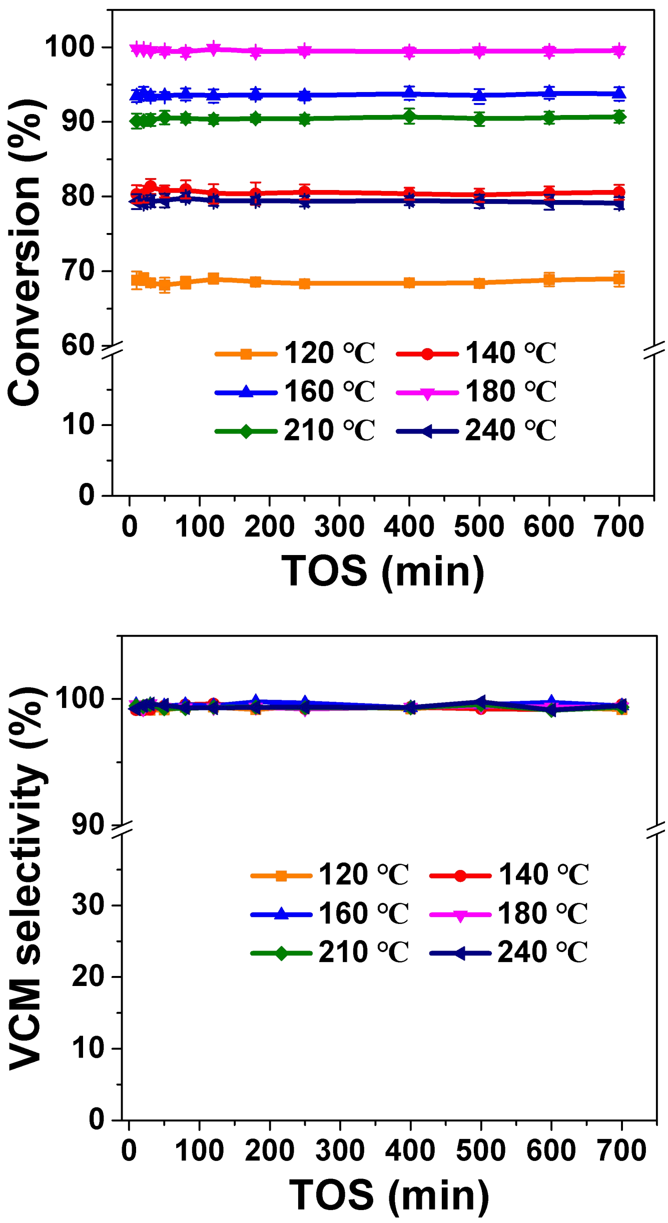

Supplementary Figure 28. Acetylene conversion efficiency and VCM selectivity of Ru-N-In/NC at different temperature (120~240 °C). The error bars indicate the standard deviations of three experimental measurements. [Reaction conditions: P = ambient pressure, Ru/In = 5.0, HCl/C_2H_2 = 1.15, and *GHSV*(C_2H_2) = 180 h^{-1}]

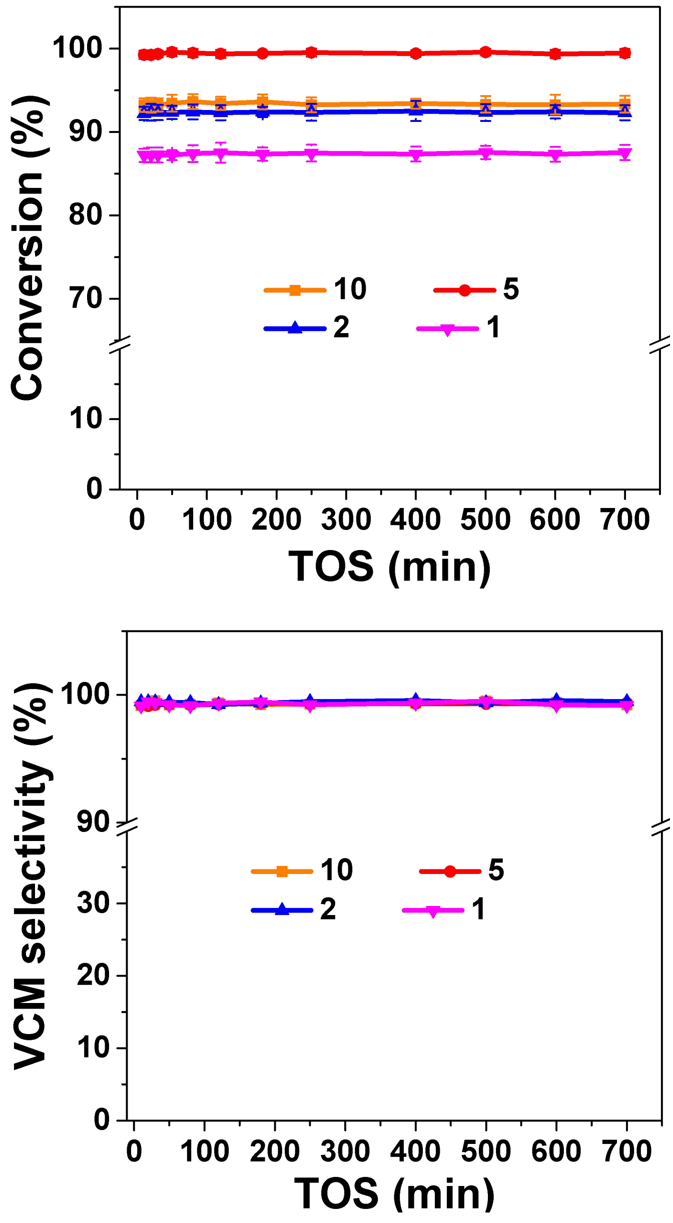

Supplementary Figure 29. Acetylene conversion efficiency and VCM selectivity of Ru-N-In/NC at different Ru/In ratios (1.0~10). The error bars indicate the standard deviations of three experimental measurements. [Reaction conditions: P = ambient pressure, T = 180 °C, HCl/C_2H_2 = 1.15, and *GHSV*(C_2H_2) = 180 h^{-1}]

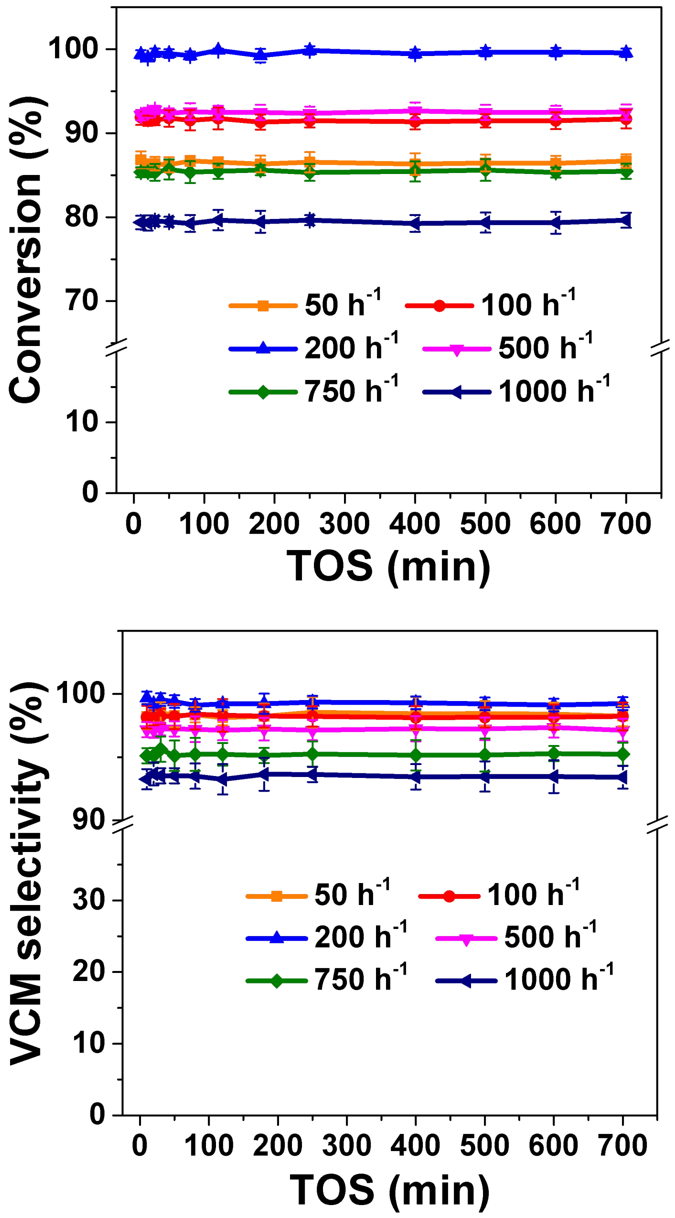

Supplementary Figure 30. Acetylene conversion efficiency and VCM selectivity of Ru-N-In/NC at different *GHSV*(C_2H_2) of 50~1000 h^{-1}. The error bars indicate the standard deviations of three experimental measurements. [Reaction conditions: P = ambient pressure, T = 180 °C, HCl/C_2H_2 = 1.15, and Ru/In = 5.0]

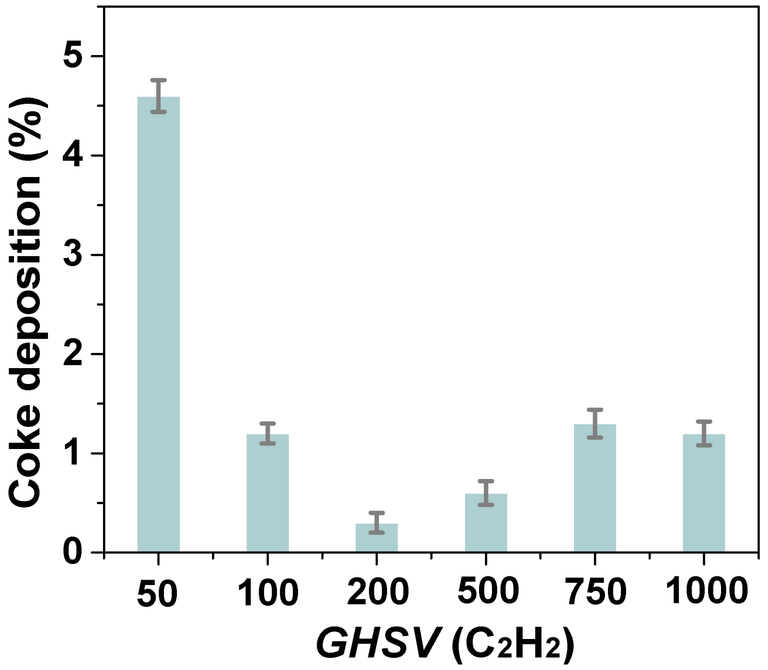

Supplementary Figure 31. Coke deposition (%), determined by thermogravimetric analysis (the amount of deposited coke is calculated from the weight loss difference for the fresh and used catalysts), of Ru-N-In/NC treated by different *GHSV*(C_2H_2) conditions. The error bars indicate the standard deviations of three experimental measurements.

A temperature of 180 °C contributes the highest C_2H_2 conversion efficiency of ~99.58%. With Ru/In ratios ranging from 0.2 to 10.0, the conversion efficiency increased first and then decreased, especially, it reached the optimum level of ~99.26% when the Ru/In ratio was 5.0. *GHSV(*C_2H_2) can exert risks on acetylene conversion by inducing coke generation. A high-level conversion efficiency (~99.38%) and lowest coke deposition (~0.3%) were achieved at *GHSV*(C_2H_2) of 180 h^{-1}. The VCM selectivity fluctuated slightly (from 99.69% to 93.26%) in the *GHSV*(C_2H_2) range of 50 to 1000 h^{-1}, causing by the production of by-products during the reaction.

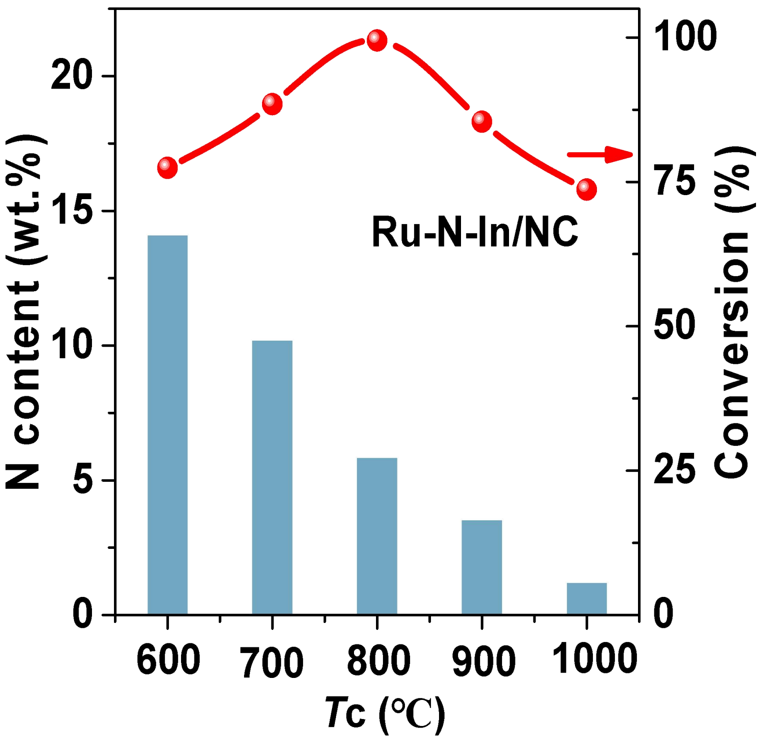

Supplementary Figure 32. Initial acetylene conversion efficiency as a function of the total N-content obtained from different pyrolysis (*T*c) carriers.

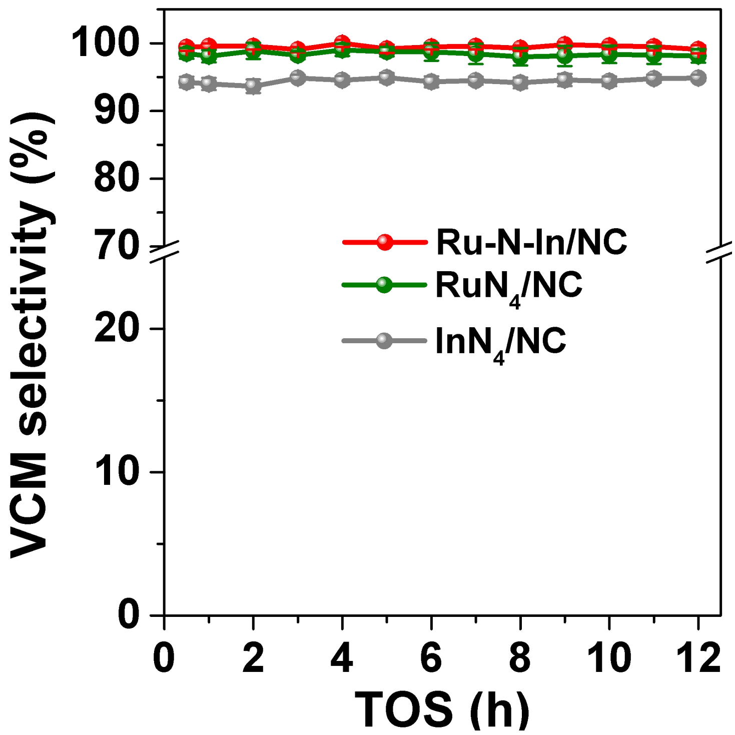

Supplementary Figure 33. VCM selectivity of Ru-N-In/NC, RuN_4/NC, and InN_4/NC. The error bars indicate the standard deviations of three experimental measurements.

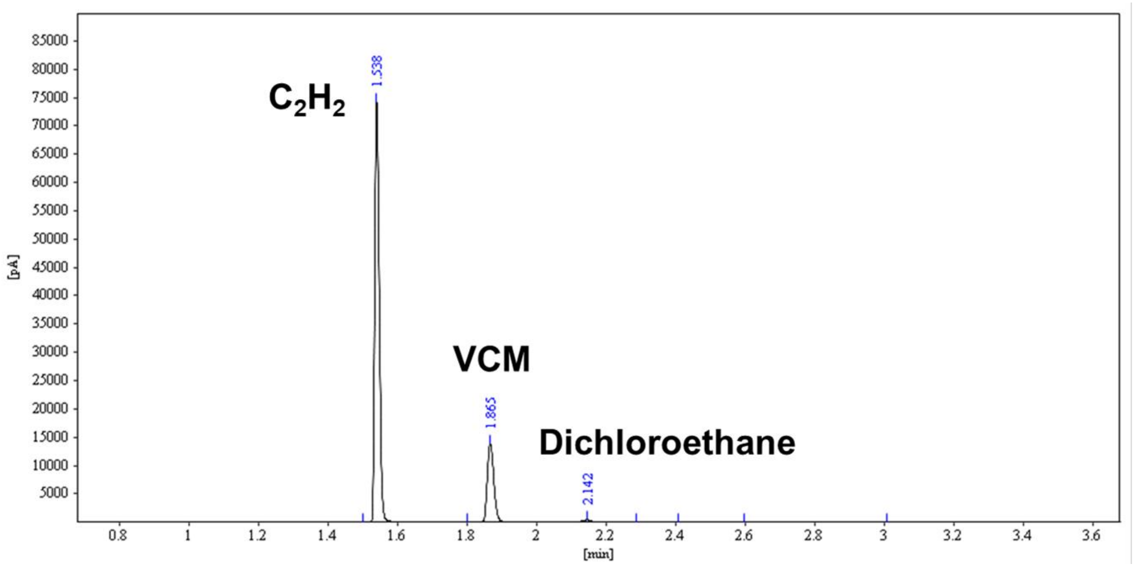

Supplementary Figure 34. The product analysis through gas chromatography over the InN_4/NC catalysts.

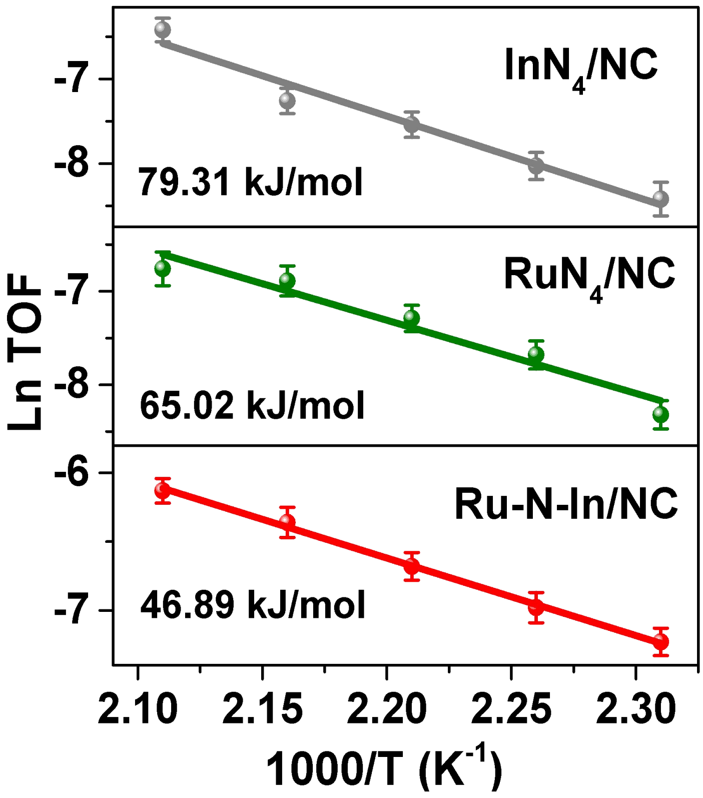

Supplementary Figure 35. Apparent activation energies (kJ/mol) of the as-prepared catalysts obtained from Arrhenius equation, in which the concentrations of C_2H_2 and HCl ranged from 20 to 40%. The error bars indicate the standard deviations of three experimental measurements.

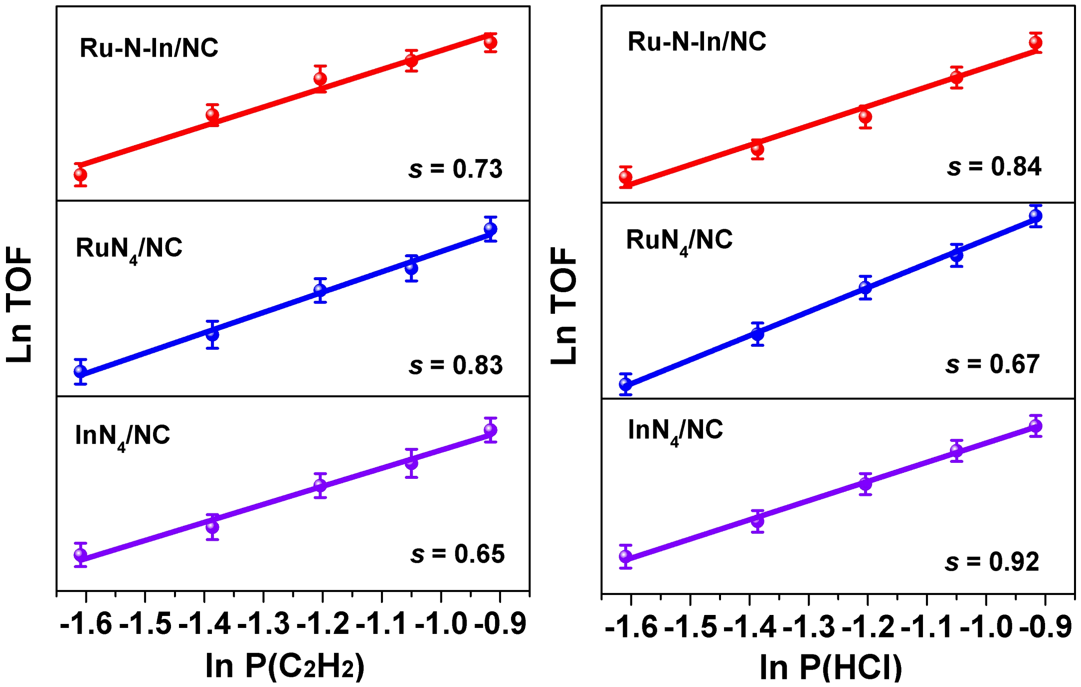

Supplementary Figure 36. Reaction orders (*s*) obtained from kinetic studies for (a) C_2H_2 and (b) HCl over Ru-N-In/NC, RuN_4/NC, and InN_4/NC. The partial reaction order of both reactants is indicated by the slope of the fitting lines. Each point was determined in an independent test to eliminate the interference of catalyst deactivation. The error bars indicate the standard deviations of three experimental measurements. [Reaction conditions: P = ambient pressure, T = 180 °C, HCl/C_2H_2 = 1.15, and the concentrations of C_2H_2 and HCl was 10 to 20%]

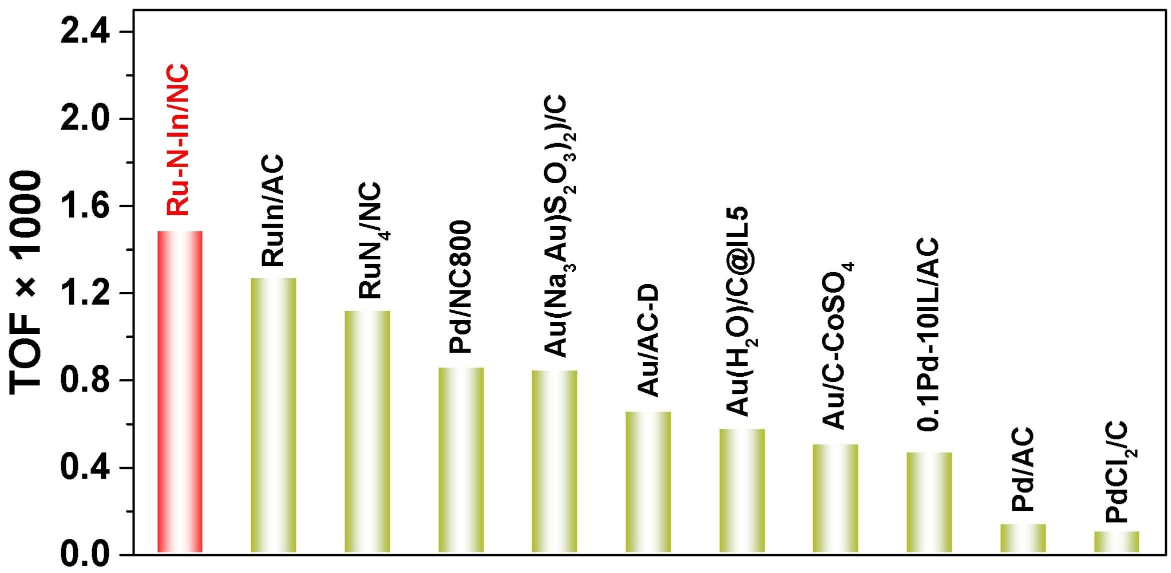

Supplementary Figure 37. Comparison of TOF (mol_{C2H2}/mol_{metal}/h) of Ru-N-In/NC with other noble metal-based catalysts. Note that all the obtained data are the same as our reaction conditions.

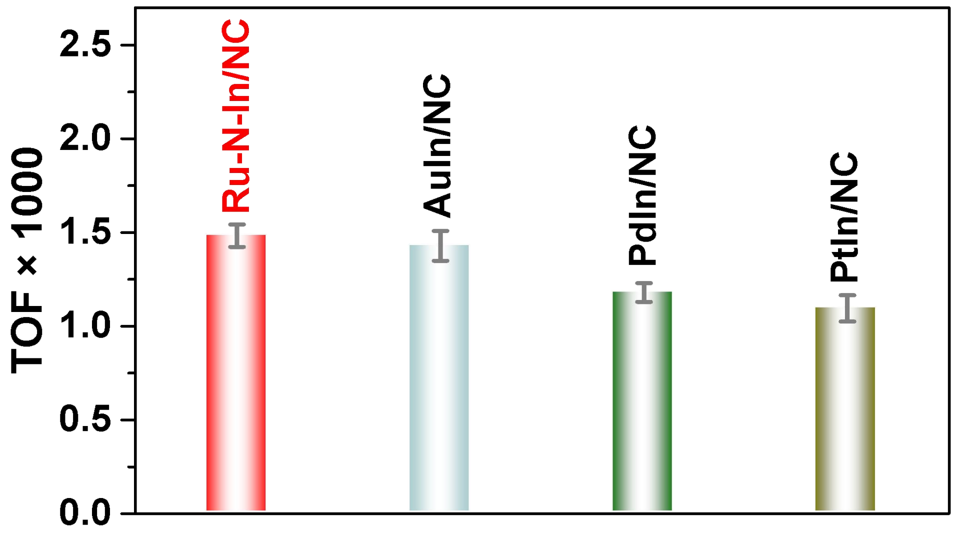

Supplementary Figure 38. Comparison of TOF ($mol_{C2H2}/mol_{metal}/h$) of Ru-N-In/NC with the AuIn/NC, PdIn/NC, and PtIn/NC catalysts. Note that the synthesis of AuIn/NC, PdIn/NC, and PtIn/NC follows the similar procedure as that of Ru-N-In/NC, shown in **Supplementary Figure 1**. The obtained data of the three samples are the same as the reaction conditions of Ru-N-In/NC. The error bars indicate the standard deviations of three experimental measurements.

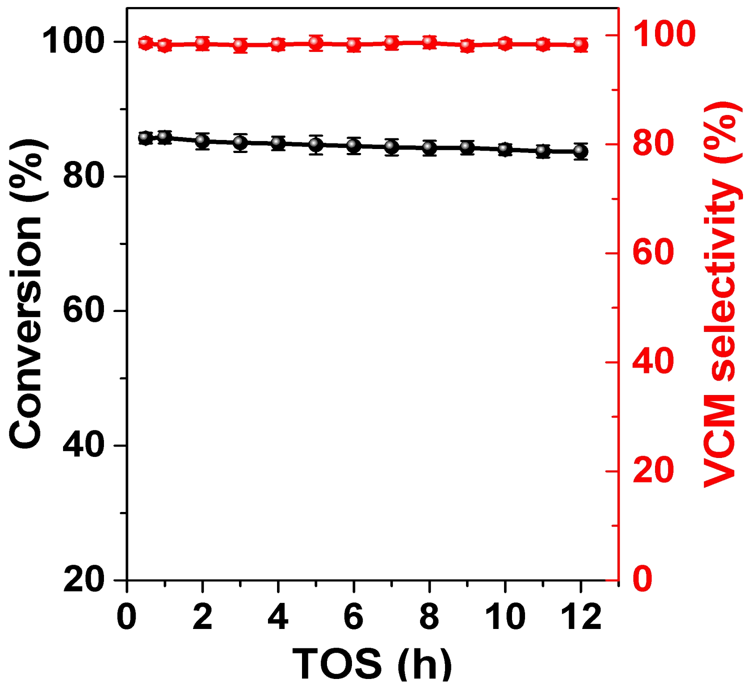

Supplementary Figure 39. Acetylene conversion efficiency and VCM selectivity for RuIn/AC. The error bars indicate the standard deviations of three experimental measurements. [Reaction conditions: P = ambient pressure, T = 180 °C, HCl/C_2H_2 = 1.15, and $GHSV(C_2H_2)$ = 180 h^{-1}].

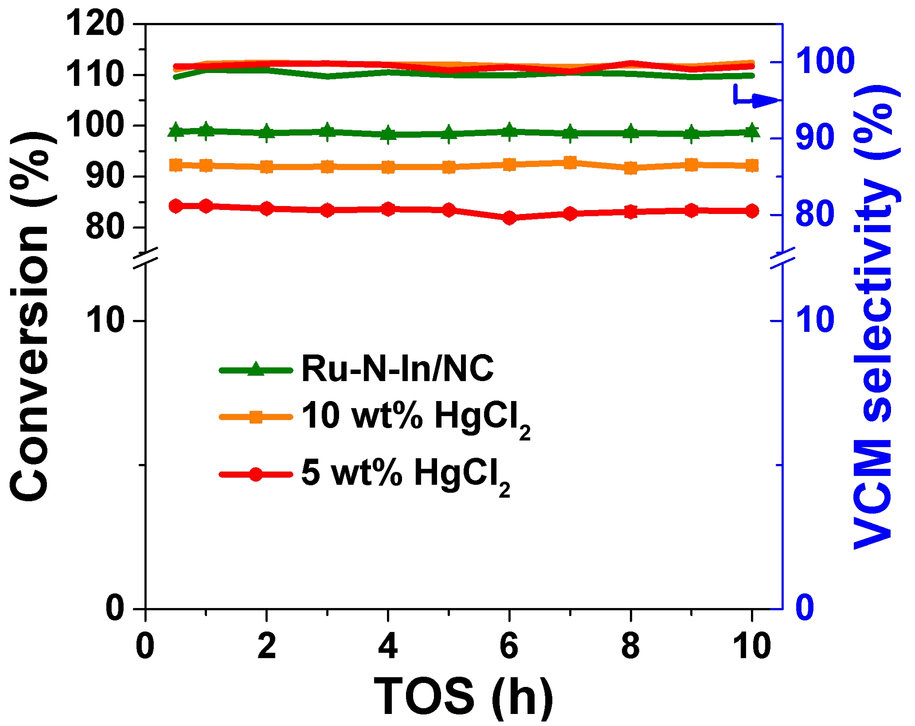

Supplementary Figure 40. Acetylene conversion efficiency and VCM selectivity for Ru-N-In/NC and $HgCl_2$/AC, where $HgCl_2$ loading was 10 wt.% (high-Hg) and 5 wt.% (low-Hg). The error bars indicate the standard deviations of three experimental measurements. [Reaction conditions: P = ambient pressure, T = 180 °C, HCl/C_2H_2 = 1.15, and *GHSV*(C_2H_2) = 180 h^{-1}].

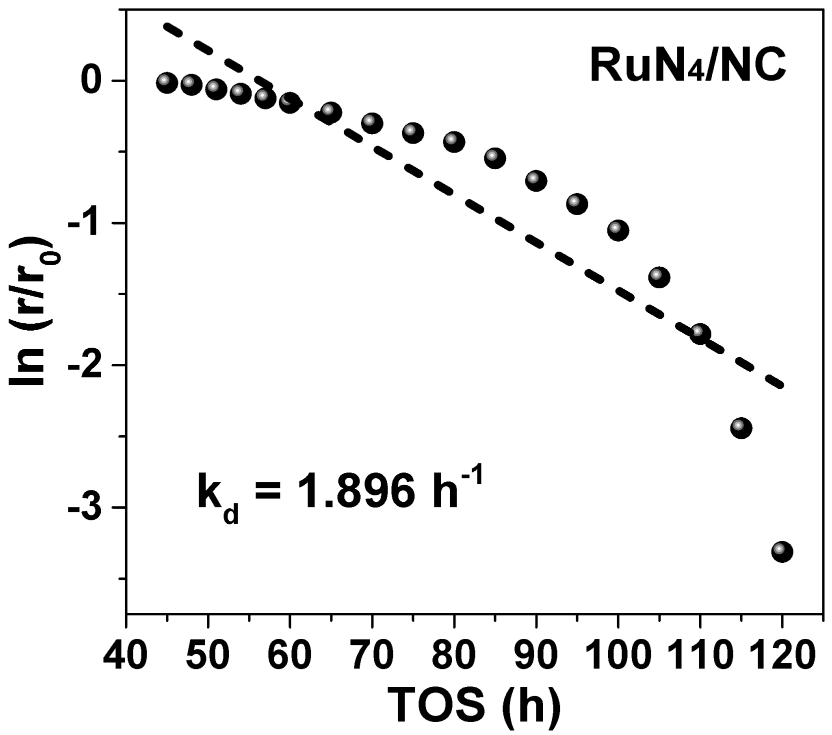

Supplementary Figure 41. Regression-fitted deactivation kinetic constant of RuN_4/NC.

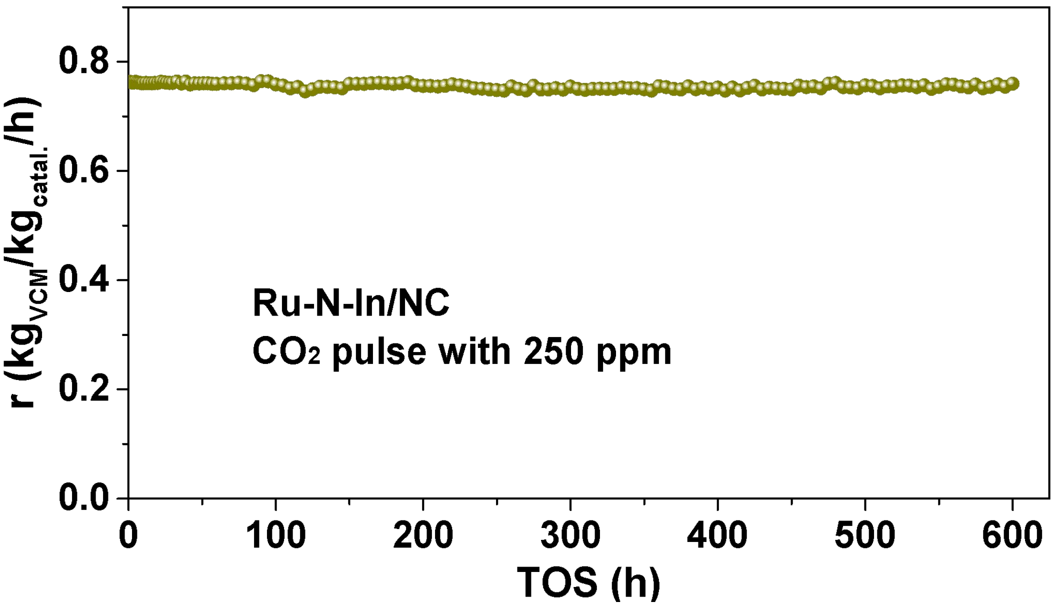

Supplementary Figure 42. Long-term catalytic performances of the Ru-N-In/NC catalyst with CO_2 pulse. [Reaction conditions: T = 180 °C, $V_{cat.}$ = 1.2 mL, CO_2 concentration = 250 ppm, P = ambient pressure, *GHSV* (C_2H_2) = 120 h^{-1}, and $V(HCl)/V(C_2H_2)$ = 1.15]

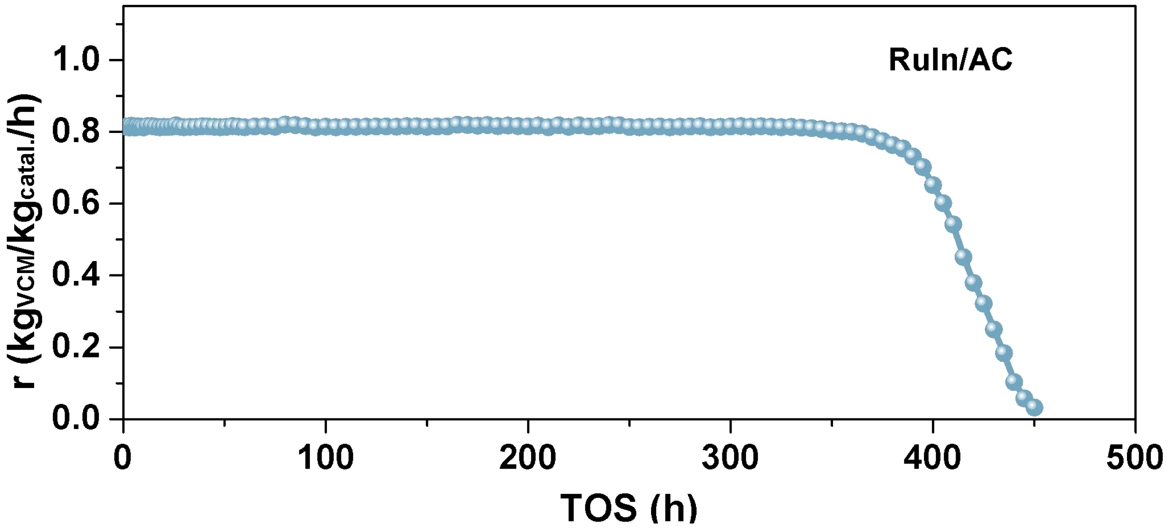

Supplementary Figure 43. Long-term catalytic performances of the RuIn/AC catalyst. [Reaction conditions: T = 180 °C, $V_{cat.}$ = 1.2 mL, P = ambient pressure, *GHSV* (C_2H_2) = 180 h^{-1}, and $V(HCl)/V(C_2H_2)$ = 1.15]

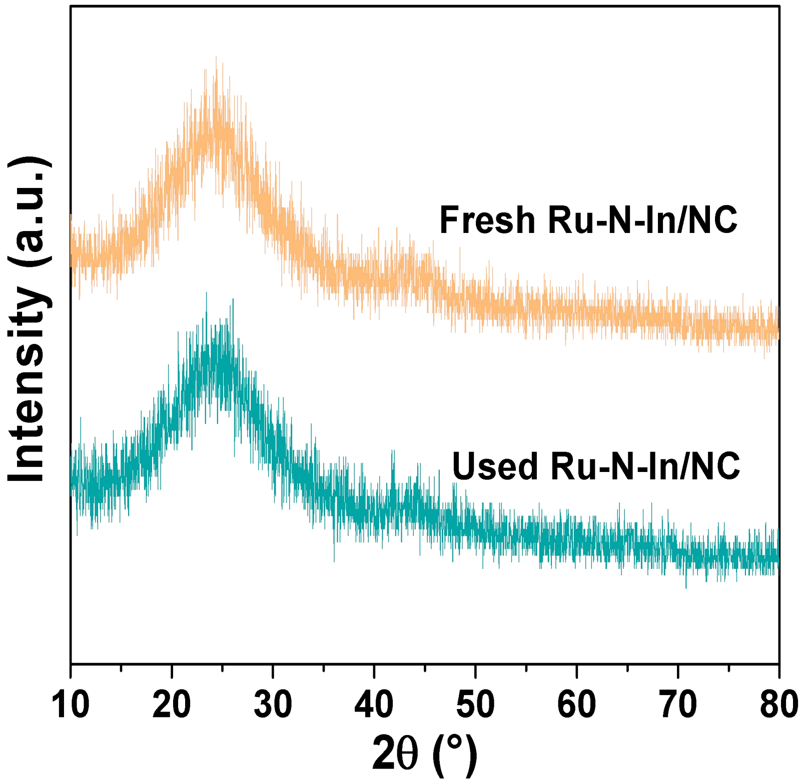

Supplementary Figure 44. XRD patterns of the fresh and used Ru-N-In/NC catalyst.

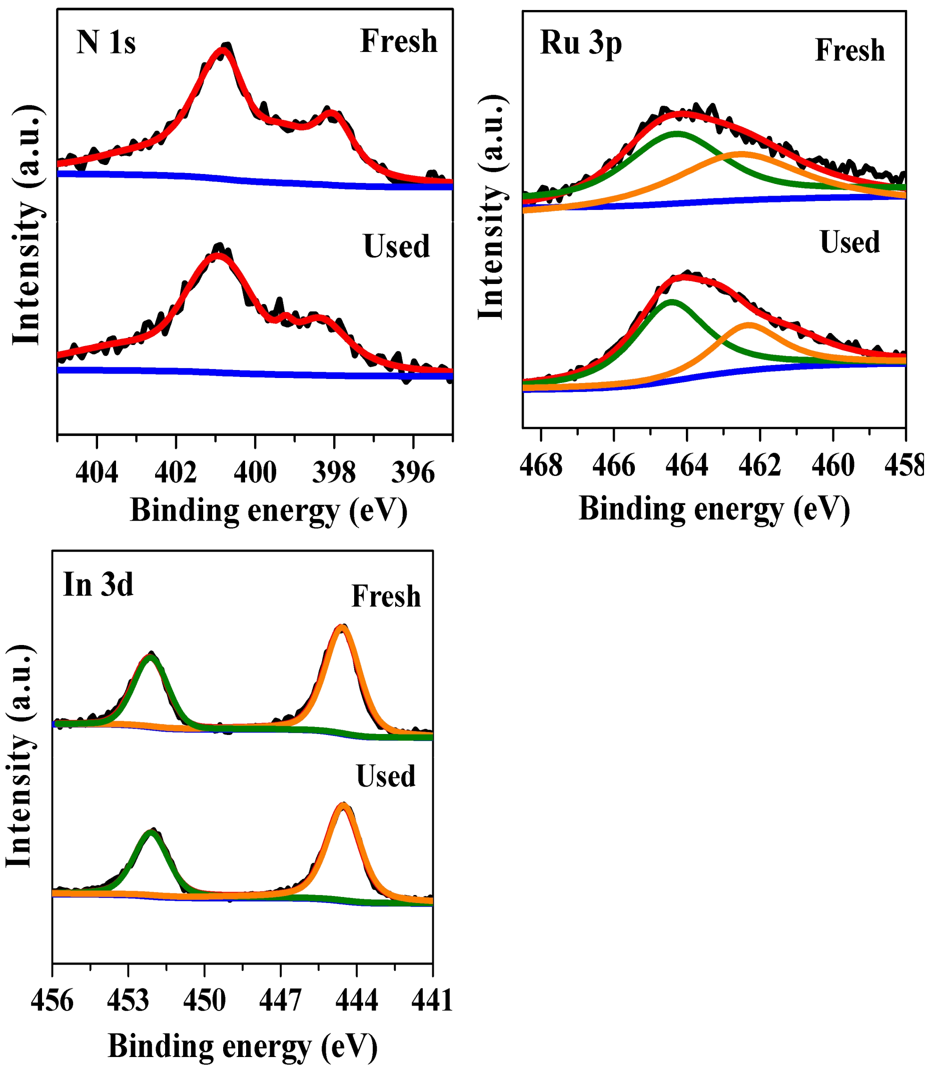

Supplementary Figure 45. XPS spectra of fresh and used Ru-N-In/NC catalyst.

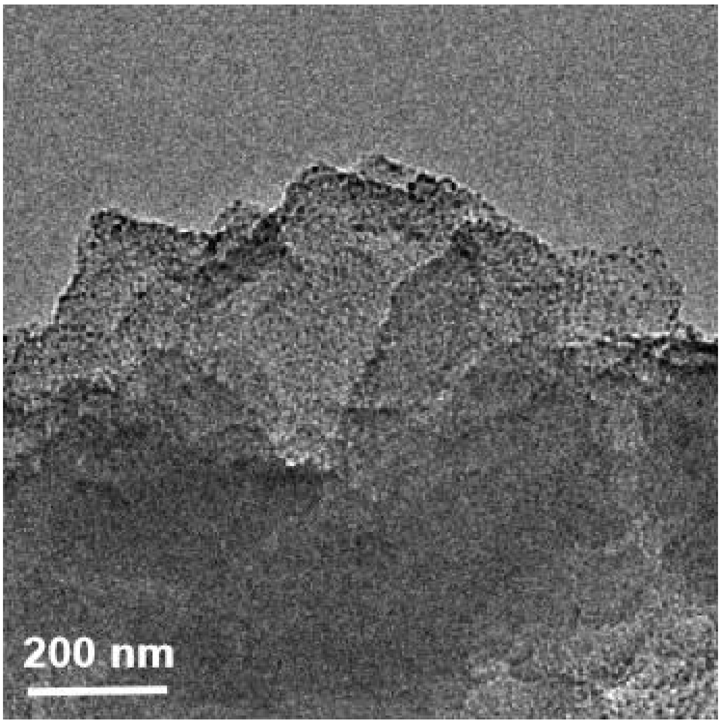

Supplementary Figure 46. TEM image of the used Ru-N-In/NC catalyst.

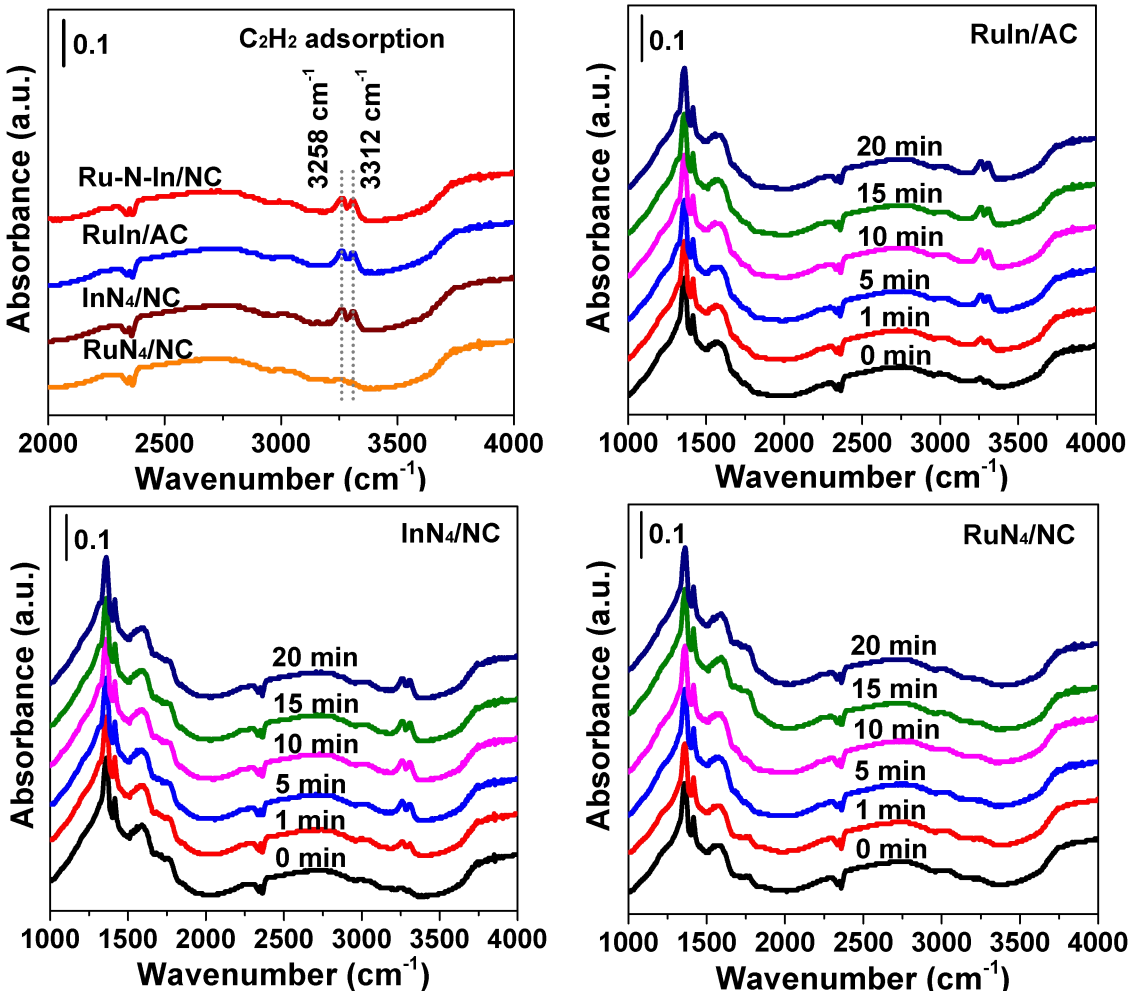

Supplementary Figure 47. In situ DRIFTS of acetylene adsorption as a function of time at 180 °C over RuIn/AC, InN_4/NC, and RuN_4/NC.

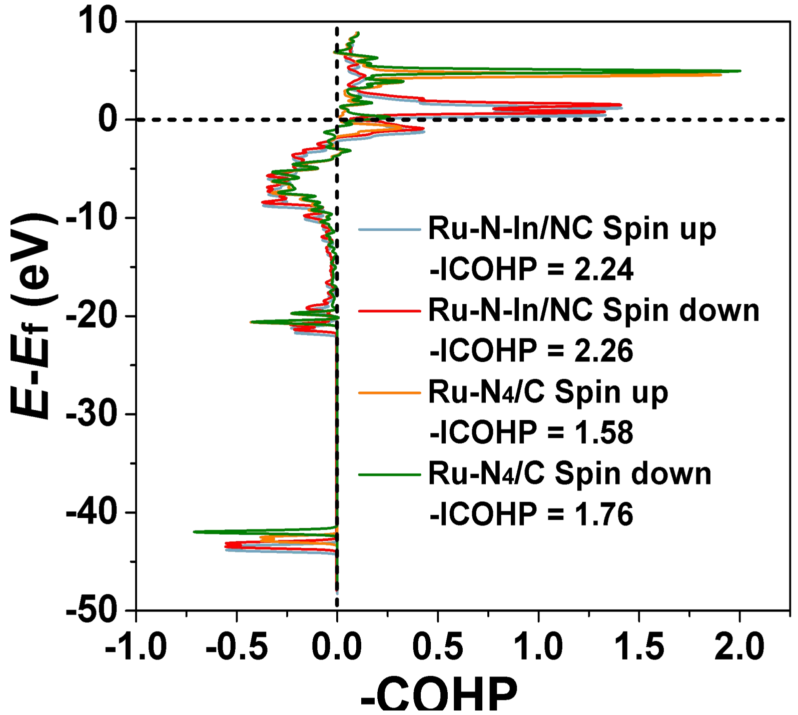

Supplementary Figure 48. COHP analysis for the adsorption of *C_2H_2 over the RuN_4/NC and Ru-N-In/NC configurations, including their respective ICOHP values.

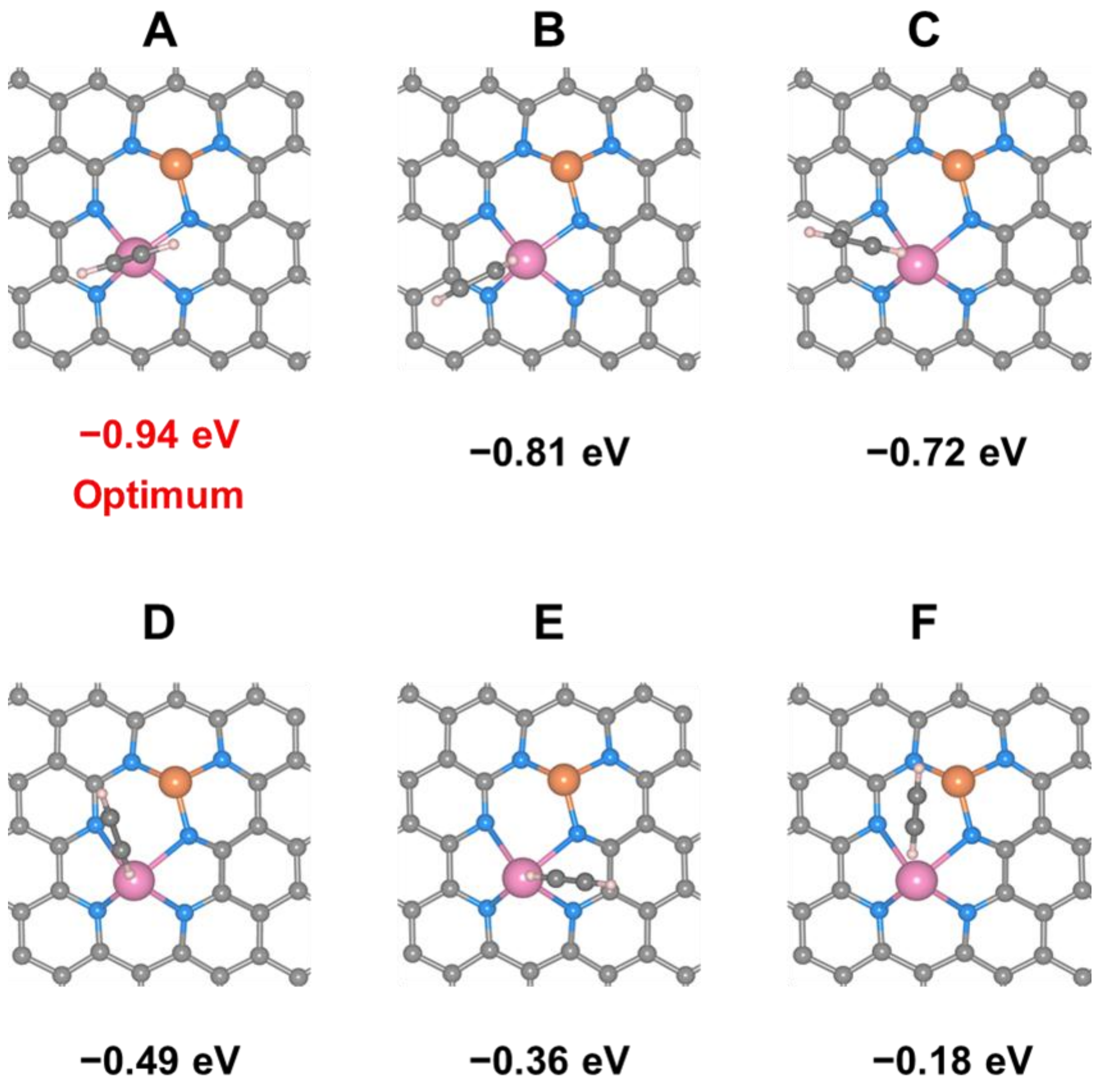

Supplementary Figure 49. Screening of the adsorption structures of the C_2H_2 molecule on Ru-N-In/NC site. The first is the optimal structure. White, gray, blue, orange, and pink balls represent H, C, N, Ru, and In atoms, respectively.

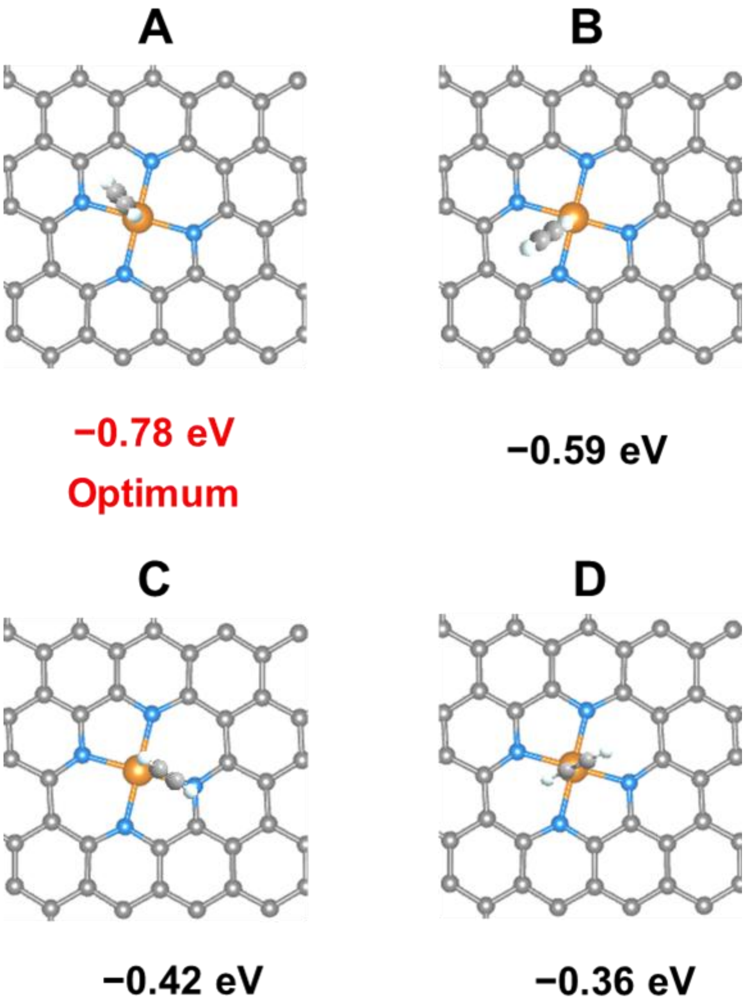

Supplementary Figure 50. Screening of the adsorption structures of the C_2H_2 molecule on RuN_4/NC site. The first is the optimal structure. White, gray, blue, green, and orange balls represent H, C, N, Cl, and Ru atoms, respectively.

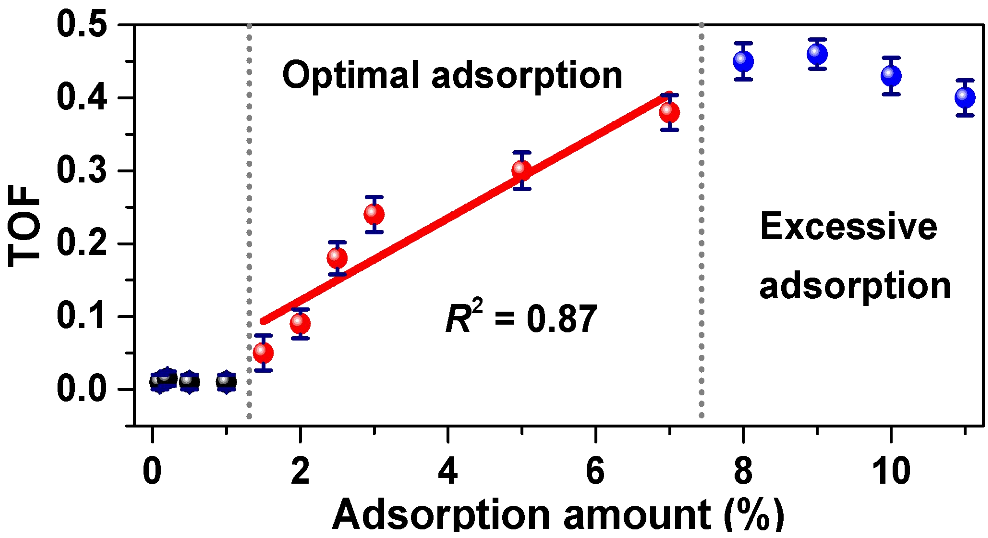

Supplementary Figure 51. The correlation between activity and optimal acetylene interaction for Ru-N-In/NC. The error bars indicate the standard deviations of three experimental measurements.

Ru-based catalysts generally require an optimum of C_2H_2 interaction to trigger reaction. According to this, the correlation between activity and optimal C_2H_2 interaction (R^2 = 0.87) was verified as the general activity descriptor for Ru-based hydrochlorination catalysts. Thus, we put forward that C_2H_2 adsorption at the active sites on Ru-N-In is the central step to initiate the catalytic cycle. For the Ru-N_4 catalyst, neither the carbon support itself, nor the Ru sites on the support leads to sufficient C_2H_2 supply. To this extent, massive unreacted or unsorbed C_2H_2 are deposited on the surface of catalyst to form coke deposits. On the contrary, the Ru-N-In/NC catalysts result in more C_2H_2 adsorption, guaranteeing the initiation of the reaction and effective activation of C_2H_2. Since Cl is preferentially coordinated to the Ru center during the reaction, the reaction coordinate can directly advance once C_2H_2 is inserted in between the Ru and Cl. This reduces the coordination and allows for the subsequent activation of HCl, followed by VCM formation by H* transfer. Conversely, the initial adsorption and activation of HCl is less favorable, as it leads to closing of the metal coordination sphere and hence compromises C_2H_2 affinity. Based on the above analysis, the introduction of In metal does not cause massive adsorption of HCl to chlorinate and deactivate Ru centers, but instead induces the orientated adsorption for C_2H_2 molecules, which then accelerates C_2H_2 activation to promote activity.

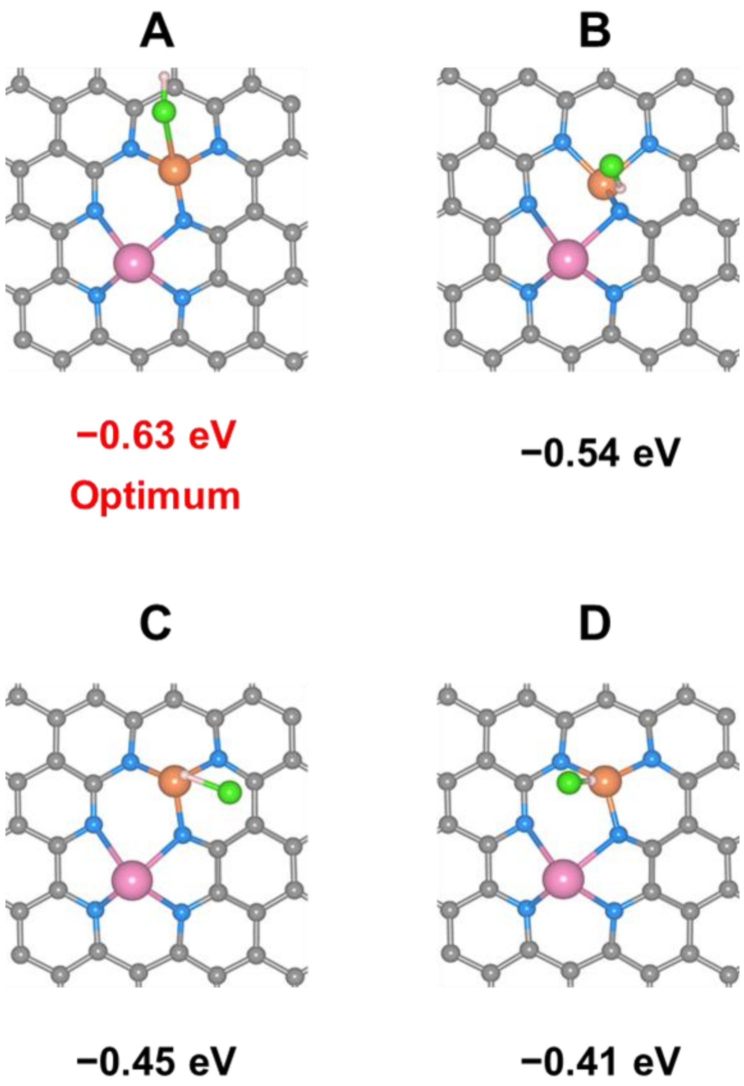

Supplementary Figure 52. Screening of the adsorption structures of the HCl molecule on Ru-N-In/NC site. The first is the optimal structure. White, gray, blue, green, orange, and pink balls represent H, C, N, Cl, Ru, and In atoms, respectively.

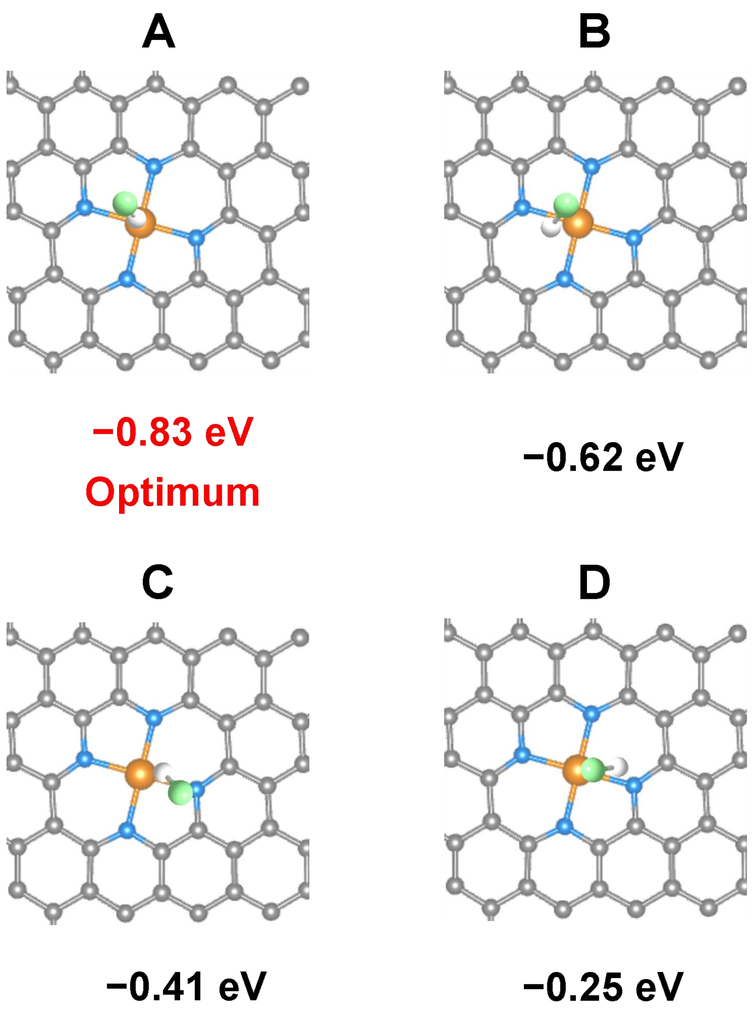

Supplementary Figure 53. Screening of the adsorption structures of the HCl molecule on RuN_4/NC site. The first is the optimal structure. White, gray, blue, green, and orange balls represent H, C, N, Cl, and Ru atoms, respectively.

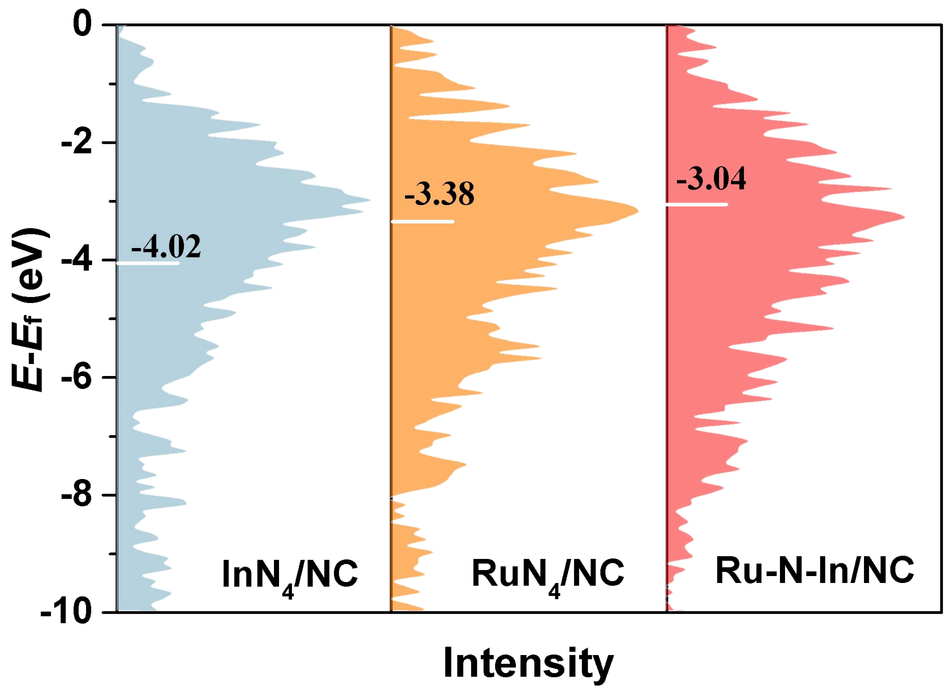

Supplementary Figure 54. Projected density of states analysis of the Ru-N-In/NC, RuN_4/NC, and InN_4/NC catalysts.

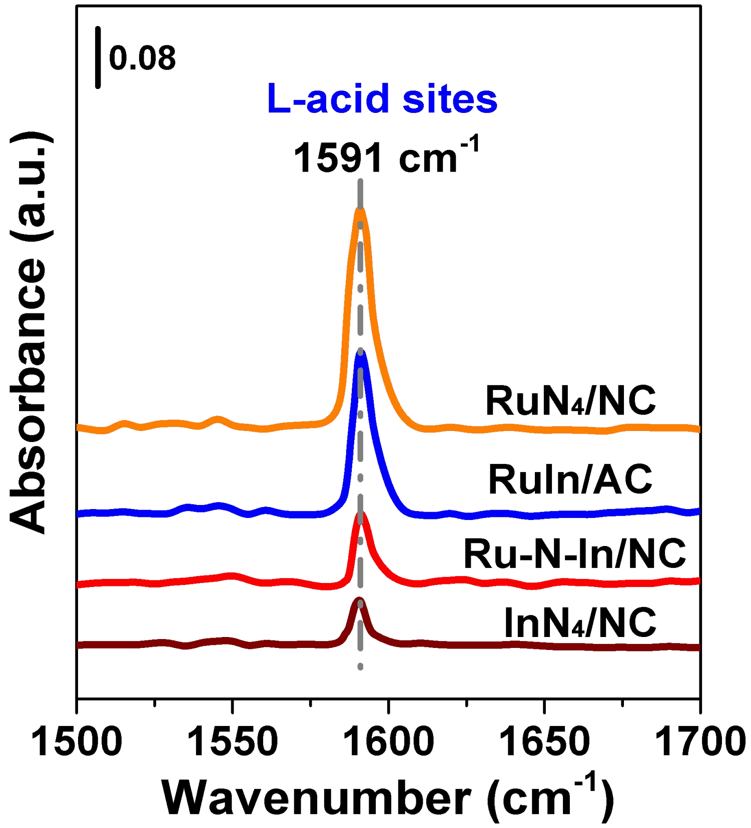

Supplementary Figure 55. NH_3-IR profiles of the Ru-N-In/NC, RuIn/AC, RuN_4/NC, and InN_4/NC catalysts.

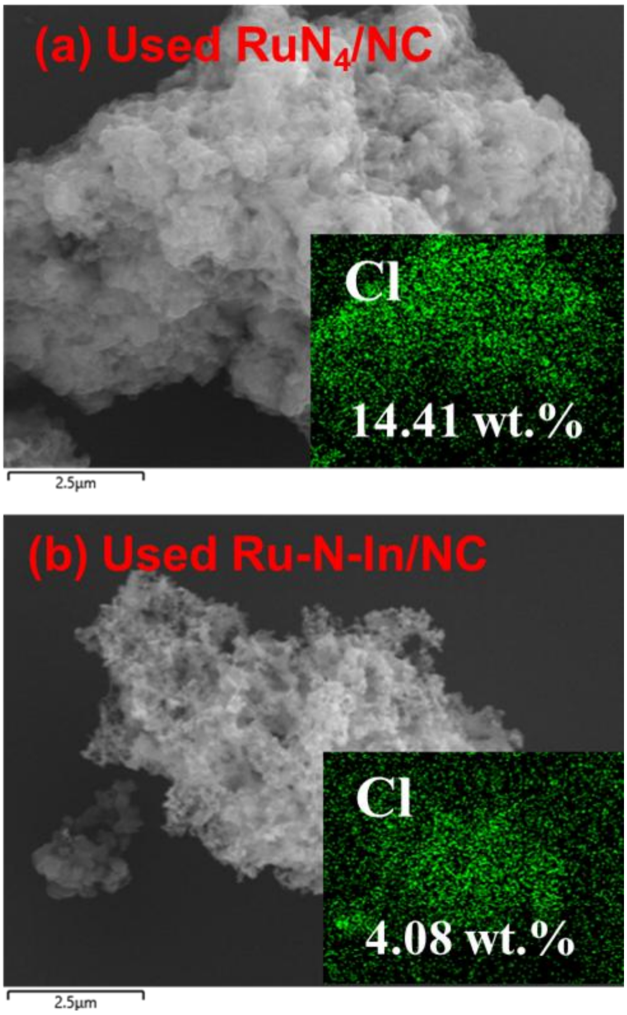

Supplementary Figure 56. SEM and EDS mapping of the used (a) RuN_4/NC and (b) Ru-N-In/NC catalysts.

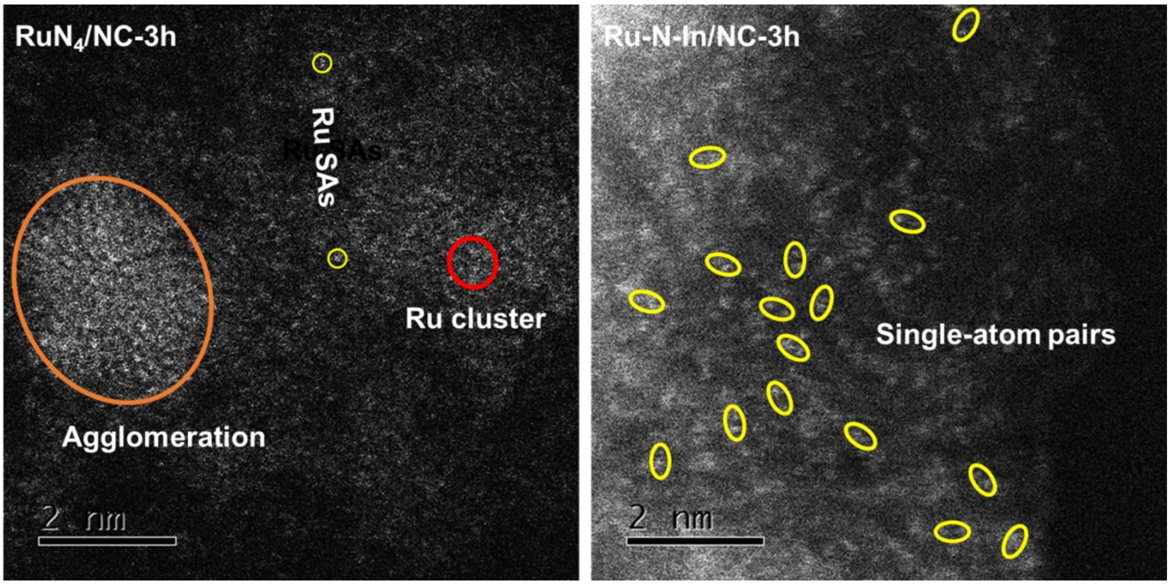

Supplementary Figure 57. AC-HAADF-STEM image of RuN_4/NC-3h and Ru-N-In/NC-3h.

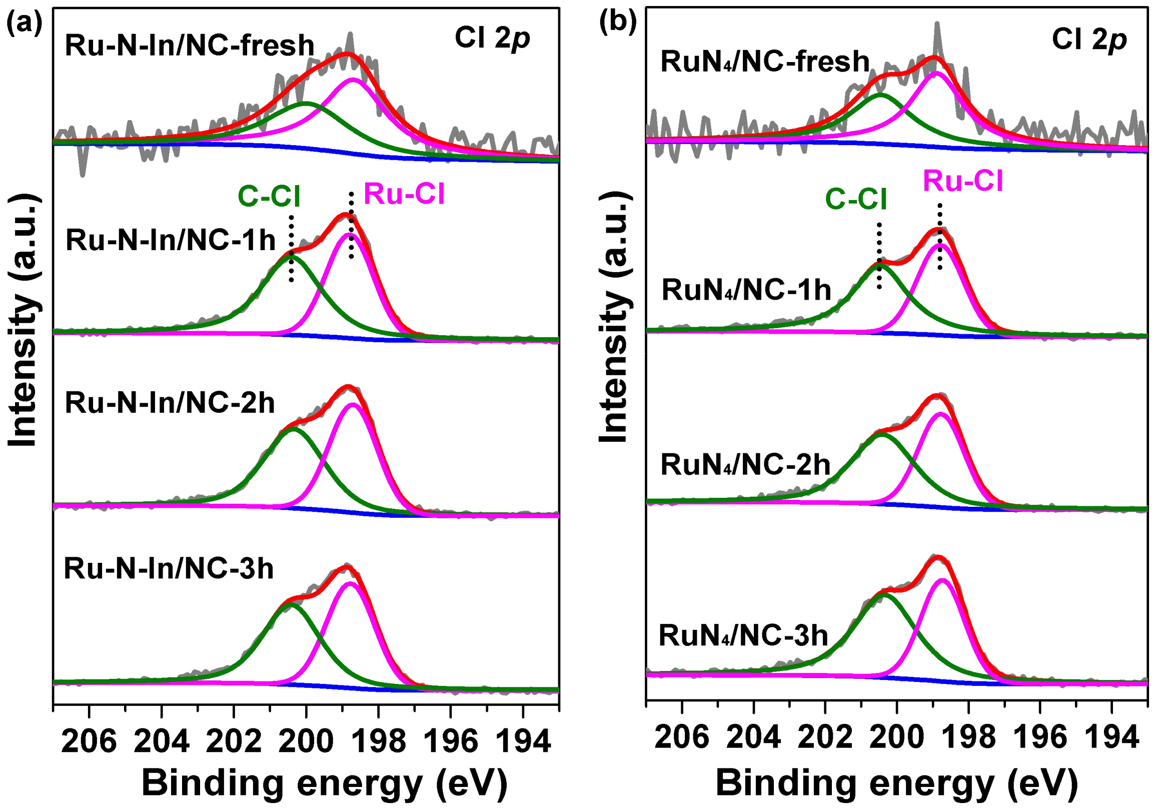

Supplementary Figure 58. Cl 2*p* XPS spectra of the fresh and progressively chlorinated (a) Ru-N-In/NC and (b) RuN_4/NC samples.

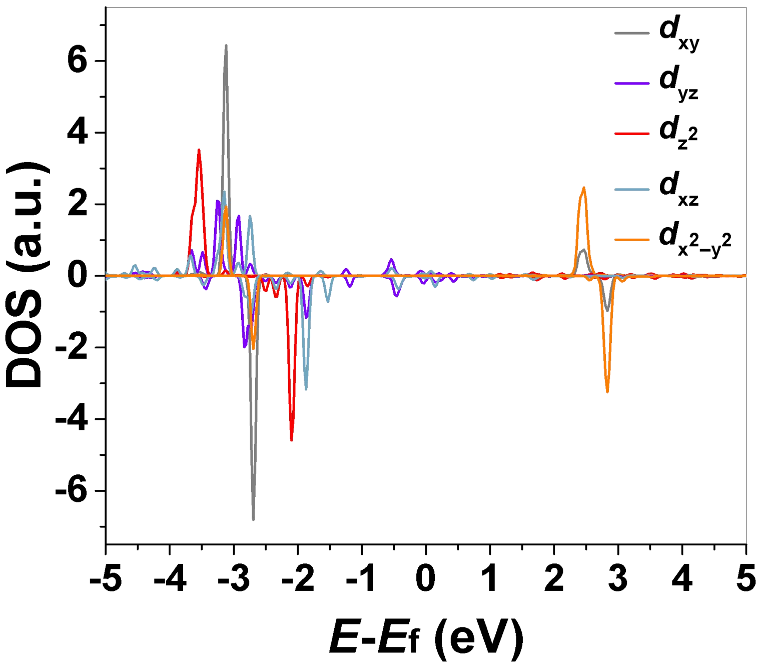

Supplementary Figure 59. Projected density of states for *d* orbitals of Ru before Cl* adsorption for RuN_4/NC.

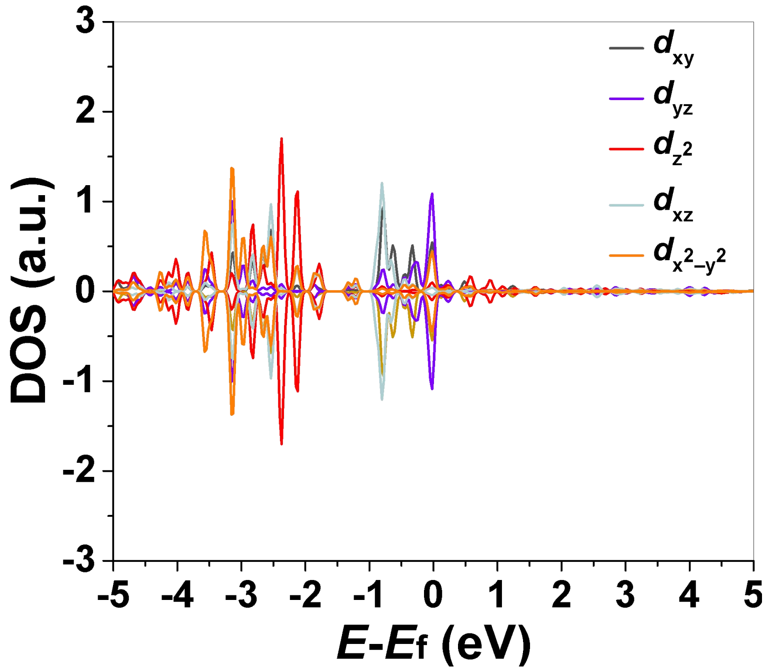

Supplementary Figure 60. Projected density of states for *d* orbitals of Ru before Cl* adsorption for Ru-N-In/NC.

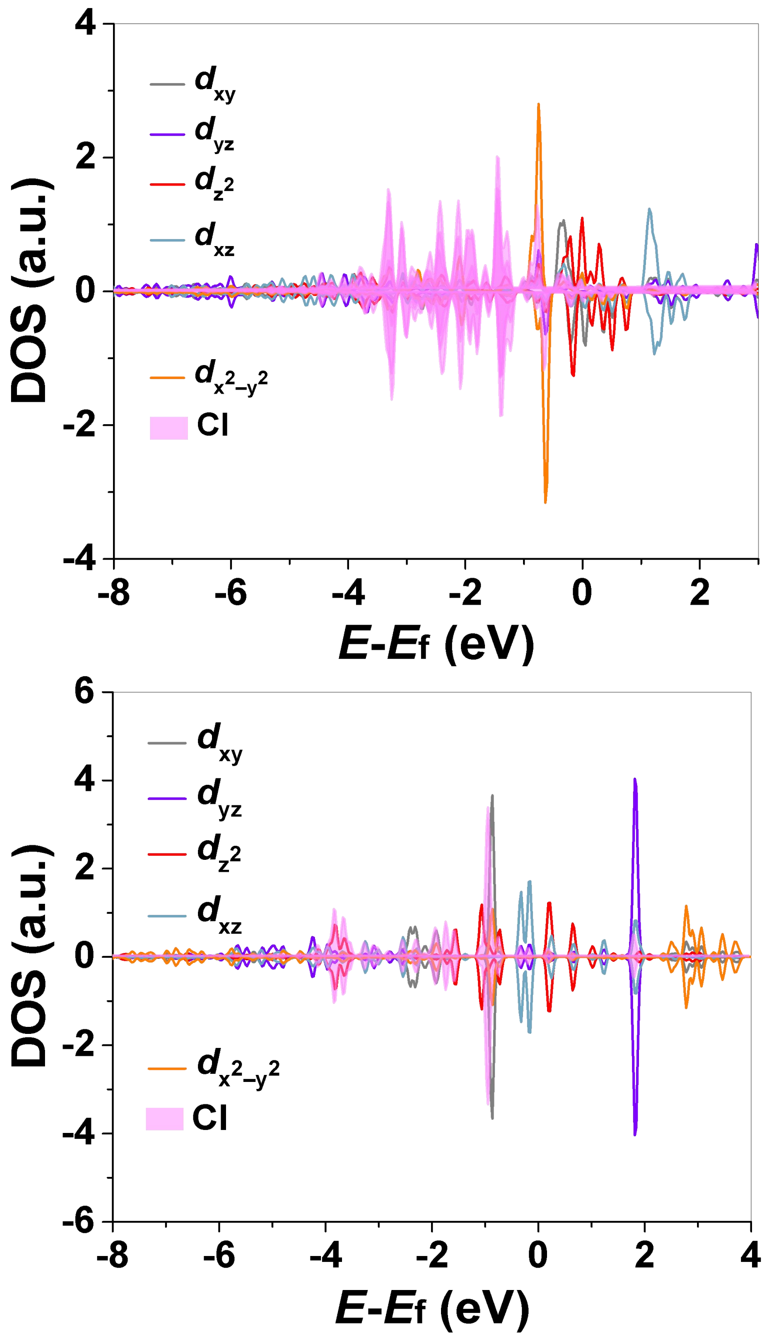

Supplementary Figure 61. Projected density of states analysis for *d* orbitals of Ru after the adsorption of the second Cl* over RuN_4/NC and Ru-N-In/NC.

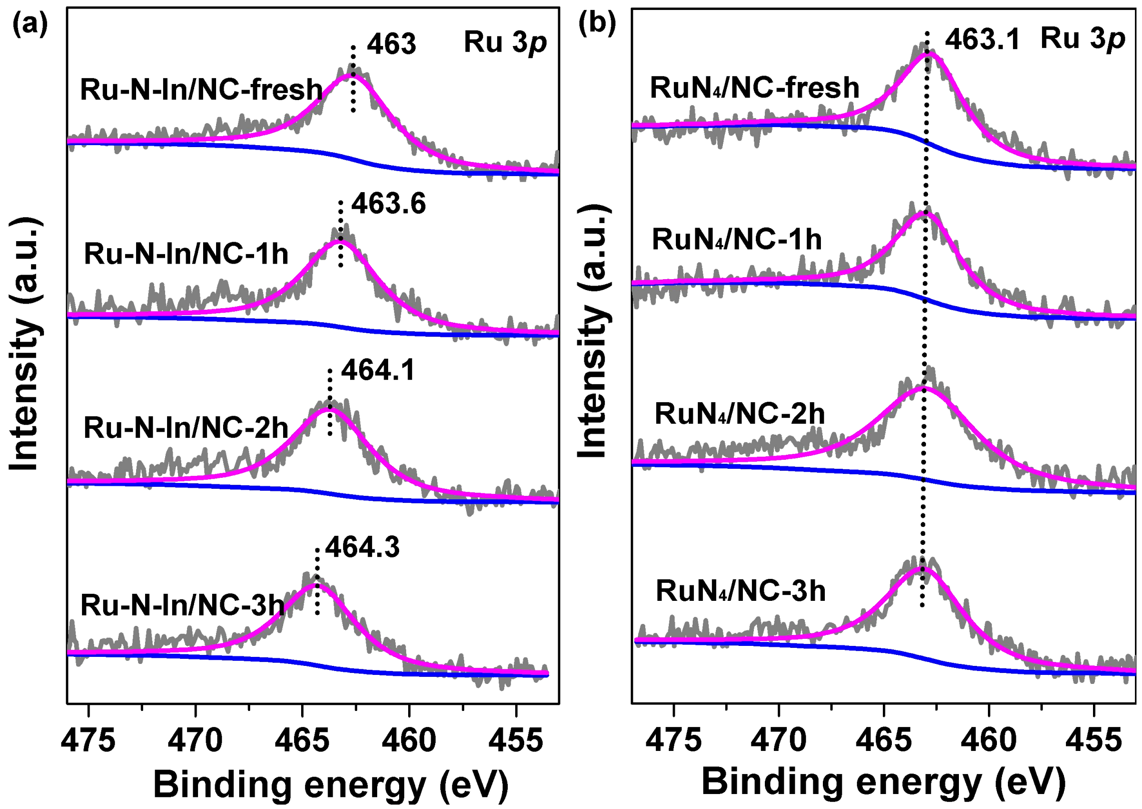

Supplementary Figure 62. Ru 3*p* XPS spectra of the fresh and progressively chlorinated (a) Ru-N-In/NC and (b) RuN_4/NC samples.

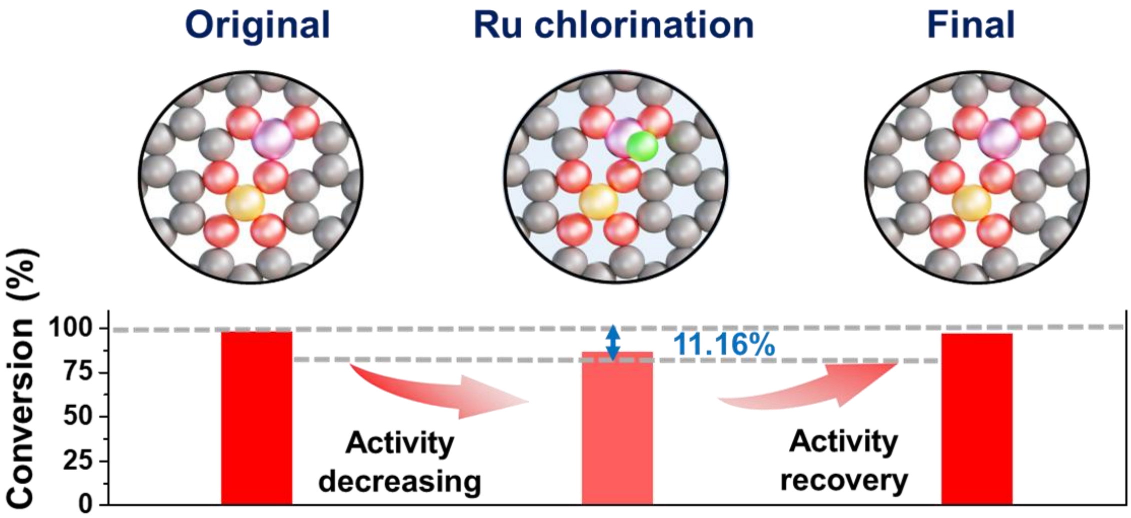

Supplementary Figure 63. Acetylene conversions and the corresponding structures of the original, chlorinated, and recovered Ru-N-In/NC catalyst. When the partial deactivation of Ru-N-In/NC catalyst occurred, we attempt to recover the single-atom Ru catalyst by warming up plus N_2 purging, where Ru-N-In/NC moiety can return to its original Ru-In single-atom pairs, and acetylene conversions of the chlorinated Ru-N-In (~87.16%) could be recovered to ~97%.

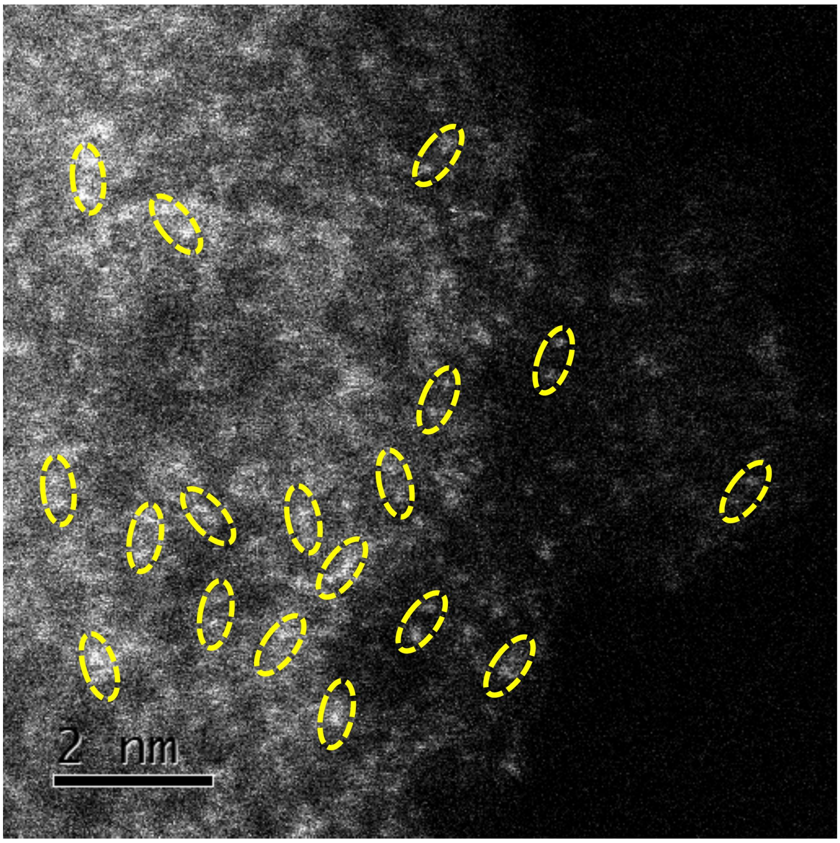

Supplementary Figure 64. AC-HAADF-STEM images of the recovered Ru-N-In/NC catalyst.

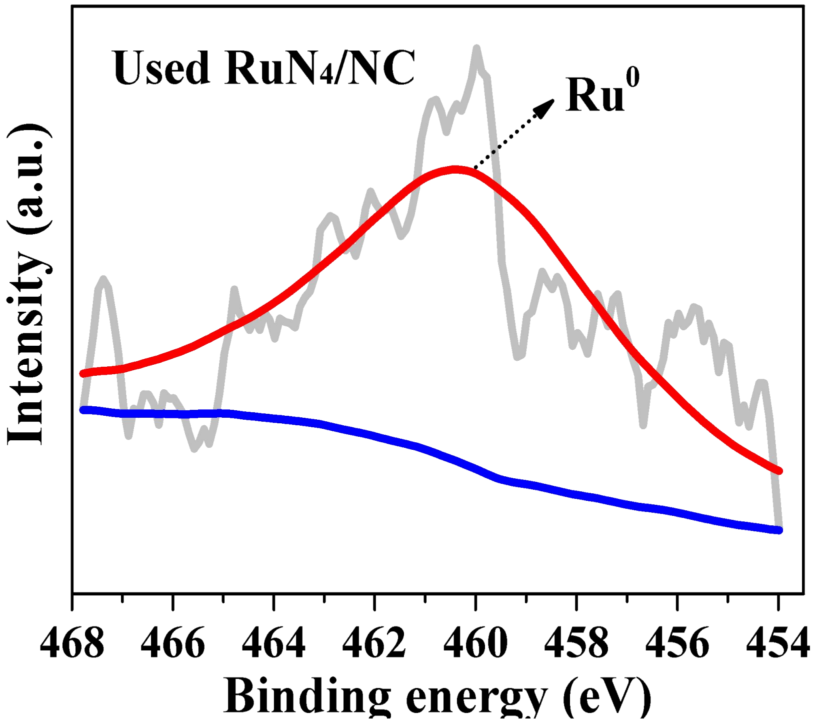

Supplementary Figure 65. The Ru 3*p* XPS spectra of the RuN_4/NC catalyst after treating by the reactive atmosphere. The reactive atmosphere here refers to the atmosphere of C_2H_2 + HCl (V(HCl)/V(C_2H_2) = 1.15) for acetylene hydrochlorination, in which the gas purities are over 99 %.

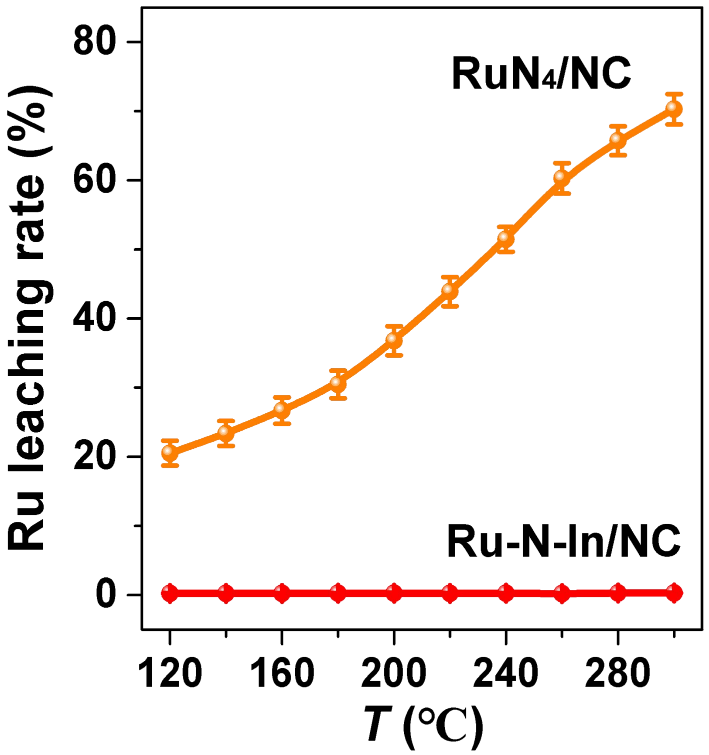

Supplementary Figure 66. Ru leaching rate of Ru-N-In/NC and RuN_4/NC with increasing temperature from 120 to 300 °C for 12 h under acetylene atmosphere. The error bars indicate the standard deviations of three experimental measurements.

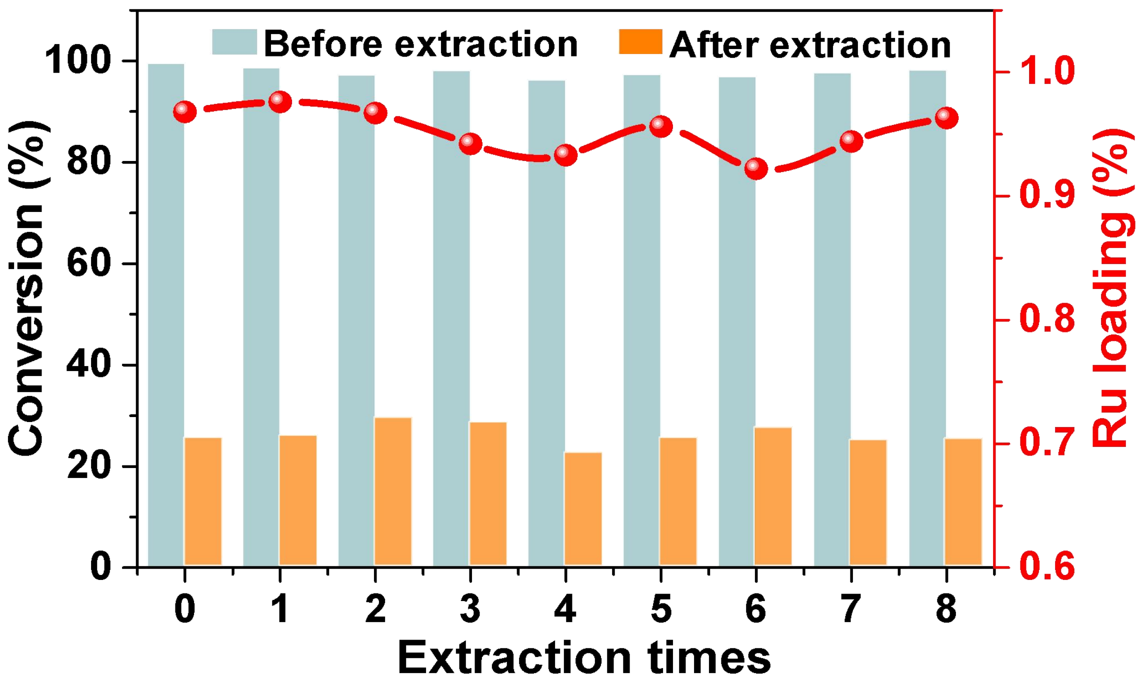

Supplementary Figure 67. Acetylene conversion efficiency of Ru-N-In/NC before and after Soxhlet extraction, and the right ordinate axis represents the actual Ru loading.

To begin with, the fresh Ru-N-In/NC catalyst was extracted in acetone solution at 120 °C for 48 h, where this process should always maintain the cooling water refluxing to prevent the escape of acetone. Then, approximately 0.25 mL of Ru extraction solution was quantified by ICP-OES, and the rest part was reloaded onto the fresh supports using the same procedures in **Supplementary Figure 1**.

Supplementary References

(1) Shang, S. et al. Highly Efficient Ru@IL/AC To Substitute Mercuric Catalyst for Acetylene Hydrochlorination. *ACS Catal.* **7,** 3510-3520 (2017).

(2) Li, J. et al. Enhanced catalytic performance of activated carbon-supported Ru-based catalysts for acetylene hydrochlorination by azole ligands. *Appl. Catal. A* **592,** 117431 (2020).

(3) Man, B. et al. Oxidation modification of Ru-based catalyst for acetylene hydrochlorination. *RSC Adv.* **7,** 23742-23750 (2017).

(4) Li, Y. et al. Improvement of imidazolium-based ionic liquids on the activity of ruthenium catalyst for acetylene hydrochlorination. *Mol. Catal.* **443,** 220-227 (2017).

(5) Li, X. et al. Synthesis of Vinyl Chloride Monomer over Carbon-Supported Tris-(Triphenylphosphine) Ruthenium Dichloride Catalysts. *Catalysts* **8,** 276 (2018).

(6) Li, X. et al. Activated Carbon-Supported Tetrapropylammonium Perruthenate Catalysts for Acetylene Hydrochlorination. *Catalysts* **7,** 311 (2017).

(7) Man, B. et al. Hydrochlorination of acetylene over the Ru-based catalysts treated by plasma under different atmospheres. *Plasma Sci. Technol.* **21,** 85501 (2019).

(8) Gu, J. et al. Hydrochlorination of Acetylene Catalyzed by an Activated Carbon-Supported Ammonium Hexachlororuthenate Complex. *Catalysts* **7,** 17 (2017).

(9) Xu, J. et al. Ultra-low Ru-promoted $CuCl_2$ as highly active catalyst for the hydrochlorination of acetylene. *RSC Adv.* 2015, **5,** 38159 (2015).

(10) Wang, B. et al. Controllable Synthesis of Vacancy-Defect Cu Site and Its Catalysis for the Manufacture of Vinyl Chloride Monomer. *ACS Catal.* **11,** 11016-11028 (2021).

(11) Fan, Y. et al. Tunable Redox Cycle and Enhanced π-Complexation in Acetylene Hydrochlorination over RuCu Catalysts. *ACS Catal.* **12,** 7579-7588 (2022).

(12) Fan, Y. et al. Metal-Organic Frameworks Encaged Ru Single Atoms for Rapid Acetylene Harvest and Activation in Hydrochlorination. *ACS Appl. Mater. Interfaces* **15,** 24701-24712 (2023).

(13) Cai, M. et al. Synthesis of a vinyl chloride monomer via acetylene hydrochlorination with a ruthenium-based N-heterocyclic carbene complex catalyst. *Catal. Sci. Technol.* **10,**

3552-3560 (2020).
(14) Zhang, M. et al. Construction of Ru-N Single Sites for Effective Acetylene Hydrochlorination: Effect of Polyethyleneimine Modifiers. *ACS Sustainable Chem. Eng.* **10**, 13991-14000 (2022).
(15) Li, J. et al. Synergistically Catalytic Hydrochlorination of Acetylene over the Highly Dispersed Ru Active Species Embedded in P-Containing Ionic Liquids. *ACS Sustain. Chem. Eng.* **8,** 10173-10184 (2020).
(16) Man, B. et al. Effect of Ru/Cl ratio on the reaction of acetylene hydrochlorination. *New J. Chem.* **41,** 14675-14682 (2017).
(17) Lan, G. et al. Direct synthesis of mesoporous nitrogen doped Ru-carbon catalysts with semi-embedded Ru nanoparticles for acetylene hydrochlorination. *Micropor. Mesopor. Mat.* **264,** 248-253 (2018).
(18) Zhang, M. et al. Ru supported on activated carbon and coated with a polydopamine layer for effective acetylene hydrochlorination. *Catal. Sci. Technol.* **12**, 4255-4265 (2022).
(19) Zhang, H. et al. Single-Atom Ruthenium Catalytic Sites for Acetylene Hydrochlorination. *J. Phys. Chem. Lett.* **12,** 7350-7356 (2021).
